# Radiation-Induced Oxidation Reactions of 2-Selenouracil in Aqueous Solutions: Comparison with Sulfur Analog of Uracil

**DOI:** 10.3390/molecules27010133

**Published:** 2021-12-27

**Authors:** Konrad Skotnicki, Ireneusz Janik, Klaudia Sadowska, Grazyna Leszczynska, Krzysztof Bobrowski

**Affiliations:** 1 Centre of Radiation Research and Technology, Institute of Nuclear Chemistry and Technology, 03-195 Warsaw, Poland; kris@ichtj.pl; 2 Notre Dame Radiation Laboratory, University of Notre Dame, Notre Dame, IN 46556, USA; 3 Institute of Organic Chemistry, Faculty of Chemistry, Lodz University of Technology, 90-924 Lodz, Poland; klaudia.sadowska@dokt.p.lodz.pl (K.S.); grazyna.leszczynska@p.lodz.pl (G.L.)

**Keywords:** 2-selenouracil, ^●^OH and ^●^N_3_ radicals, 2c-3e Se∴Se-bonded intermediates, pulse radiolysis, time-resolved conductivity, TD-DFT methods

## Abstract

One-electron oxidation of 2-selenouracil (2-SeU) by hydroxyl (^●^OH) and azide (^●^N_3_) radicals leads to various primary reactive intermediates. Their optical absorption spectra and kinetic characteristics were studied by pulse radiolysis with UV-vis spectrophotometric and conductivity detection and by the density functional theory (DFT) method. The transient absorption spectra recorded in the reactions of ^●^OH with 2-SeU are dominated by an absorption band with an λ_max_ = 440 nm, the intensity of which depends on the concentration of 2-SeU and pH. Based on the combination of conductometric and DFT studies, the transient absorption band observed both at low and high concentrations of 2-SeU was assigned to the dimeric 2c-3e Se-Se-bonded radical in neutral form (2^●^). The dimeric radical (2^●^) is formed in the reaction of a selenyl-type radical (6^●^) with 2-SeU, and both radicals are in equilibrium with K_eq_ = 1.3 × 10^4^ M^−1^ at pH 4 (below the pK_a_ of 2-SeU). Similar equilibrium with K_eq_ = 4.4 × 10^3^ M^−1^ was determined for pH 10 (above the pK_a_ of 2-SeU), which admittedly involves the same radical (6^●^) but with a dimeric 2c-3e Se-Se bonded radical in anionic form (2^●−^). In turn, at the lowest concentration of 2-SeU (0.05 mM) and pH 10, the transient absorption spectrum is dominated by an absorption band with an λ_max_ = 390 nm, which was assigned to the ^●^OH adduct to the double bond at C5 carbon atom (3^●^) based on DFT calculations. Similar spectral and kinetic features were also observed during the ^●^N_3_-induced oxidation of 2-SeU. In principle, our results mostly revealed similarities in one-electron oxidation pathways of 2-SeU and 2-thiouracil (2-TU). The major difference concerns the stability of dimeric radicals with a 2c-3e chalcogen-chalcogen bond in favor of 2-SeU.

## 1. Introduction

The 200th anniversary of the discovery of selenium by the “father of Swedish chemistry”, Jöns Jakob Berzelius, was celebrated in 2017. Nowadays, over 200 years later, interest in selenium compounds in the scientific community is growing rapidly. In recent years, nearly 2000 scientific papers have been published every year regarding the chemical and biological significance of selenium. Selenium compounds play a wide variety of key roles in the functioning of living organisms. This topic was discussed in several reviews [1,2,3,4,5,6,7,8].

The most important organic selenium compound found in living organisms is selenocysteine (CysSeH), which is a component of over 25 various proteins [9]. CysSeH was found in the active site of the glutathione peroxidase family [10] and is essential for the activity of this enzyme. Selenium compounds found in the active center of enzymes are usually more reactive than their sulfur counterparts.

One of the biochemically most relevant differences between sulfur- and selenium-based compounds, incorporated into the active site of enzymes as cysteine (CysSH) or CysSeH, is the prototropic equilibrium. At physiological pH (7.4), the thiol group of CysSH exists in a protonated form (p*K*_a_ of CysSH ⇄ CysS^−^ + H^+^ equilibrium is equal to 8.4). On the other hand, the much lower p*K*_a_ of CysSeH ⇄ CysSe^−^ + H^+^ equal to 5.2 results in the existence in organisms predominantly in the form of selenolate anions (CysSe^−^) [11]. This is due to the weaker bond to the hydrogen atom, together with the increase in size and the polarizability of selenium. In spite of their lower basicity, CysSe^−^ are more nucleophilic than thiolates (CysS^−^), presumably due to the higher polarizability of selenium than sulfur [12], with a polarizability volume of 3.8 Å for Se compared to 2.9 Å for S [13]. In general, this difference results in a much larger nucleophilicity of CysSeH compared to CysSH at a physiological pH, as selenols are completely converted to CysSe^−^, whereas thiols only slightly exist in the form of CysS^−^ [1]. Therefore, CysSe^−^ is, for example, much more reactive with hydroperoxides and disulfides [14]. Selenides are also more nucleophilic than sulfides [15].

The greatest divergence between selenium and sulfur chemistry takes place in the redox reactions involving these two elements, irrespectively whether they are one-electron or two-electron oxidation reactions [1]. For instance, the rates of two-electron oxidation of sulfides to sulfoxides and selenides to selenoxides are fairly comparable, with selenium being more reactive while the second two-electron oxidation of selenoxides to selenone is much slower. This is in part due to the much higher dipolar character of the Se-O bond, resulting in lower nucleophilicity of the lone pair on the Se atom. Two-electron oxidation of selenols to a selenenic acid is presumably also faster than the similar oxidation of a thiol to sulfenic acid. This might be in part a consequence of the bond dissociation energy (BDE) of the O–H bond in a selenic acid (81.2 kcal mol^−1^), which is higher than a Se–H bond (78.9 kcal mol^−1^). The opposite trend was reported for sulfur, the BDE for the O–H bond in a sulfenic acid (68.6 kcal mol^−1^), which is weaker than the BDE for the S–H bond (87.6 kcal mol^−1^) [16]. The difference in one-electron reduction potentials of selenium compounds vs. sulfur compounds was clearly revealed using a specific one-electron oxidant, i.e., azide radical (N_3_^●^). This radical characterized by the standard reduction potential of the ^●^N_3_/N_3_^−^ redox couple E^0^ = 1.33 V vs. NHE [17] being able to oxidize selenourea (Seu) [18] and selenomethionine (SeMet) [19], unlike thiourea (Su) [20] and methionine (Met) [21]. This indicates that the standard reduction potentials of the Seu^●+^/Seu and SeMet^●+^/SeMet redox couples are lower than the standard reduction potentials of the Su^●+^/Su and Met^●+^/Met redox couples. Indeed, the reduction potential of the SeMet^●+^/SeMet redox couple was determined to be 1.21 V vs. NHE at pH 7 [19], which is lower than the reduction potential of the Met^●+^/Met redox couple equal to 1.48 V vs. NHE, and measured by cyclic voltammetry [22]. This latter fact is also confirmed by the efficient repair of tyrosyl (TyrO^●^) and tryptophyl (TrpN^●^) radicals by selenols. The rate constants for the reaction of substituted TyrO^●^ radicals with CysSeH and selenoglutathione (Se-Glu) are in the range (5–8) × 10^8^ M^−^ s^−1^. In contrast, CysH and glutathione (Glu) react three and five orders of magnitude slower than their selenium analogs [23].

The mechanisms and kinetics of reactions involving selenium radicals formed by the reaction of ^●^OH radicals and other one-electron oxidants with selenium-containing amino acids and their derivatives were quite extensively studied by pulse radiolysis. There have been several pulse radiolysis studies regarding the oxidation of SeU [24,25,26], SeMet [19,24,26,27], methylselenocysteine [24], CysSeH [26,28], and selenocystine [24,29,30] and its derivatives, selenocystamine and diselenodipropionic acid [29].

For instance, the oxidation of SeMet by ^●^OH radicals follows a similar mechanism as in the case of Met. At a pH less than 3, the Se∴OH adduct is converted to a selenium-centered radical cation (SeMet^●+^), which reacts with another parent SeMet, forming an intermolecularly three-electron bonded dimeric radical cation (MetSe∴SeMet)^+^ [24]. The nature and spectral behavior of (MetSe∴SeMet)^+^ are similar to analogous dimeric radical cation reported for methionine. However, the equilibrium constant (*K*) for the formation of (MetSe∴SeMet)^+^ is (Equation (1)):

MetSe^●+^ + Met Se ⇆ (MetSe∴SeMet)^+^ (1)

Equal to 9.2 × 10^3^ M^−1^ is higher as compared to the equilibrium constant (*K*) formation of (MetS∴SMet), which is equal to 1.9 × 10^3^ M^−1^. Similarly, as for Met, ^●^OH-induced oxidation at a neutral pH leads to intramolecular three-electron bonded Se∴N radical cation (SeMet(S∴N)^+^). However, the lifetime of SeMet(S∴N)^+^ is substantially longer (τ_1/2_ = 70 μs) [31], in comparison to analogous Met(S∴N)^+^ (τ_1/2_ = 200 ns) [32].

Since SeMet^●+^ is a weaker oxidant than its sulfur analog (vide supra), a decarboxylation reaction leading to the respective α-amino radical is less efficient in SeMet. The yield of released CO_2_ from SeMet(Se∴N)^+^ is nearly half of that released from Met(S∴N)^+^ [31].

Some attention was also devoted to time-resolved studies of radicals derived from selenocysteine CysSeH [28,33,34]. The difference in one-electron reduction potentials of a selanyl radical (RSe^●^) (E^0^ (RSe^●^/RSeH/H^+^) = + 0.43 V vs. NHE) and a thiyl radical (RS^●^) (E^0^ (RS^●^/RSH/H^+^) = + 0.92 V vs. NHE) were nicely illustrated by their reactions with tyrosine (Tyr) and tryptophan (Trp). Thiyl radicals (RS^●^) are capable of oxidizing Tyr and Trp to the respective tyrosyl (TyrO^●^) and tryptophyl radicals (TrpN^●^), while RSe^●^ radicals are not [33]. The RSe^●^ radicals also have a much lower ability to abstract hydrogen from the ^α^C-H bond in the protein backbone in comparison to RS^●^ radicals. The estimated intramolecular reaction rate for such reaction was found to be extremely low (0.1 s^−1^) [34], in comparison to an analogous reaction involving RS^●^ radicals (10^5^ s^−1^) [35]. This is in line with the respective values of BDE of Se–H (310 kJ mol^−1^), S–H (365 kJ mol^−1^), and ^α^C–H (330–365 kJ mol^−1^) bonds since the formation of RSe^●^ radical requires less energy than the formation of RS^●^ and > ^α^C^●^ radicals [28].

The observed distinct differences in redox chemistry of selenium-centered radicals and radicals’ cations in comparison to analogous sulfur ones in peptides and proteins were recently reviewed [31].

Selenium-substituted nucleobases also exist naturally in living organisms in the form of 2-selenouridine derivatives in amino-acid-transfer ribonucleic acids (t-RNAs) [36,37]. They were found in position 34 (the wobble position) of the anticodon stem loop, only in tRNA iso-acceptors specific for glutamate, glutamine, and lysine [38]. Their function in tRNA was attributed to their different base-pairing affinity in the anticodon loop of the tRNA in comparison to uridine and 2-thiouridine [39]. Such Se-derivatization on tRNA probably improves the accuracy and efficiency of protein translation [40].

An interesting aspect related to the difference in redox properties of selenium and sulfur in substituted nucleobases is related to their reactions with H_2_O_2_. The only data currently available concern the oxidative transformation of 2-selenouracil-5-carboxylic acid (2-SeU-5-COOH) [41] and 2-selenouracil 2-SeU [42] using hydrogen peroxide (two-electron oxidant). The redox chemistry of 2-SeU-5-COOH and 2-SeU were compared with the sulfur analogs by identification of stable products. In the first paper, it was shown that oxidation of 2-SeU-5-COOH resulted in a diselenide intermediate, followed by conversion to seleninic acid. In contrast, 2-TU-5-COOH oxidation led to oxidized intermediates (sulfenic, sulfinic, and sulfonic acids) followed by irreversible desulfurization of the latter [41]. In the second paper, the results obtained by the authors clearly demonstrate that 2-SeU undergoes much easier two-electron oxidation than 2-thiouracil (2-TU), forming predominantly in the first step, diselenide U-Se-Se-U, which spontaneously rearranges via single deselenation to Se-containing two-ring compound. The latter compound, after the loss of H_2_Se, forms the two-ring product, which, in turn, reacts with the remaining diselenide to yield a three-ring product. Unlike 2-SeU, oxidation of 2-TU predominantly leads to uracil [42]. These results confirmed significantly different oxidation pathways of 2-SeU and 2-TU [41,42]. Based on the oxidation studies of 2-thiouridine [43], it was suggested that desulfurization of 2-thiouridine may be a part of a redox signaling pathway in response to oxidative stress [44]. Therefore, the substitution of sulfur with selenium might have serious consequences connected with the alteration of the signaling pathway [41].

On the other hand, there are virtually no data describing the reactivity of selenium derivatives of nucleic acid bases with biologically important radicals, such as hydroxyl radicals (HO^●^) and superoxide radical anions (O_2_^●−^), which are involved in one-electron redox reactions during oxidative stress. The current paper is dedicated to the extension of our studies on the oxidation of the chalcogen analogs of uracil. We report in this paper our in-depth studies on one-electron oxidation of 2-selenouracil (2-SeU) induced by ^●^OH and ^●^N_3_ radicals in aqueous solutions. The influence of 2-SeU concentration, pH, and the character of one-electron oxidant on the transient absorption spectra and the kinetics transient was addressed and compared with the respective spectra and kinetics of radicals and radical ions observed in the sulfur analog of uracil (2-TU). The presence of a selenium atom (instead of an oxygen atom) at position C2 in 2-SeU may result (as in the case of 2-TU) in the establishment of additional tautomeric equilibria. Quantum mechanical calculations using the DFT method showed that 2-SeU behaves similarly to 2-TU in an aqueous environment. The tautomerism activation barriers were found to be high enough (though the energy gaps are smaller than for 2-TU) to conclude that only the oxo-selenone structure will be found in the gas phase as well in a solution [45]. The absorption spectra of potential transients produced in ^●^OH–induced oxidation of 2SeU together with the relative energy profiles for the H abstraction and ^●^OH addition from/to neutral 2-SeU, respectively, were calculated using the DFT method.

## 2. Results

### 2.1. Oxidation of 2-SeU by ^●^OH Radicals

#### 2.1.1. Influence of 2-SeU Concentration and pH on Absorption Spectra

**Figure 1:**
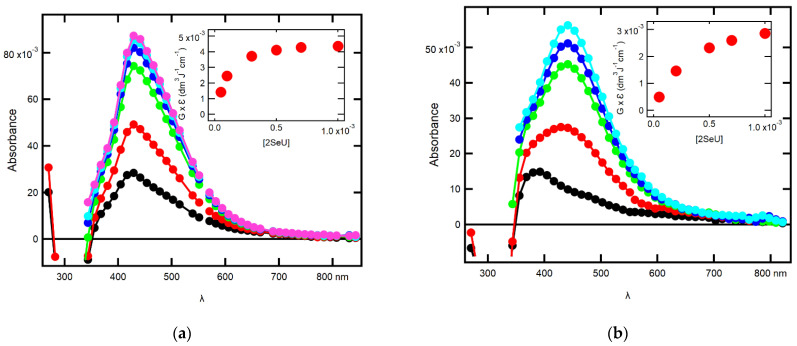
Transient absorption spectra recorded in N_2_O-saturated unbuffered aqueous solutions: (**a**) at pH 4 containing 0.05 mM (●), 0.1 mM (●), 0.3 mM (●), 0.5 mM (●), 0.7 mM (●), 1 mM (●) of 2-SeU recorded 7.8 μs, 5.4 μs, 2.5 μs, 1.6 μs, 1.2 μs, and 1 μs, respectively, after electron pulse; (**b**) at pH 10 containing 0.05 mM (●), 0.2 mM (●), 0.5 mM (●), 0.7 mM (●), and 1 mM (●) of 2-SeU recorded 6 μs, 3 μs, 2 μs, 1.6 μs, and 1.3 μs, respectively after electron pulse. Insets: maximum absorbances (expressed as G × ε) recorded at λ = 440 nm in a function of 2-SeU concentration.

Taking into account, reported in our earlier work, the effect of 2-thiouracil (2-TU) concentration on the transient absorption spectra [46], we decided to record UV-vis transient absorption spectra at various 2-SeU concentrations to check whether and to what extent concentration of 2-SeU affects spectral characteristics. The spectral changes observed after pulse irradiation of N_2_O-saturated unbuffered aqueous solutions at two pHs (4 and 10) containing 2-SeU in the concentration range of 0.05 mM to 1 mM are shown in Figure 1.

The recording times are selected at the maximum of the absorbance measured at λ = 440 nm after electron pulse for the specified concentration of 2-SeU. It is clearly seen that the absorption spectra recorded at pH 4 for the concentration range 0.5 mM–1 mM of 2-SeU are dominated by the absorption band with λ_max_ = 440 nm, with nearly the same intensities expressed as G × ε_440_ (Figure 1a, inset). For the remaining three lower concentrations of 2-SeU (0.05, 0.1, and 0.3 mM), the intensities of absorption bands are substantially lower (Figure 1a, inset); however, their shapes and positions of the absorption maxima are only slightly different in comparison to the absorption spectra recorded for the higher concentrations (Figure 1a). On the other hand, the spectral changes observed at pH 10 clearly show that for the lowest concentration of 2-SeU (0.05 mM), the absorption spectrum is dominated by the broad and relatively weak absorption band with λ_max_ = 390 nm. For the four-fold higher concentration of 2-SeU (0.2 mM), the maximum of the absorption band is red-shifted by ~50 nm, and for 10-fold and higher concentrations of 2-SeU, the absorption spectra are dominated again by the absorption band with λ_max_ = 440 nm (Figure 1b). This is a strong indication that some other products are formed, and their spectral contribution is revealed only at the lowest concentration of 2-SeU, in particular at pH = 10.

It has to be noted that the intensities of the absorption bands with λ_max_ = 440 nm at the highest concentration of 2-SeU vary considerably with pH. The intensity of the absorption band recorded in solutions at pH 4 is higher by nearly 50% in comparison to pH 10 (insets in Figure 1).

Based on our earlier work on 2-TU [46], the transient absorption spectrum with λ_max_ = 440 nm was tentatively assigned to the formation of dimeric radical with 2c-3e selenium-selenium bond. In principle, by analogy to 2-TU, the formation of the ^●^OH adducts at C5 and C6 positions leading to the respective C-centered radicals can also be expected. However, their possible spectral contribution, even at a lower concentration of 2-SeU, was only clearly revealed at pH 10. For higher concentrations of 2-SeU, their absorption bands are probably hidden under the absorption band assigned earlier to the dimeric selenium radicals/radical anions at both pHs. More experimental evidence for their formation, if any, should be obtained by studying the time evolution of absorption spectra, especially at short times, when the absorption band assigned to the dimeric selenium radicals/radical anions was not fully developed.

#### 2.1.2. Influence of pH on the Time Evolution of the Absorption Spectra at Low Concentrations of 2-SeU

**Figure 2:**
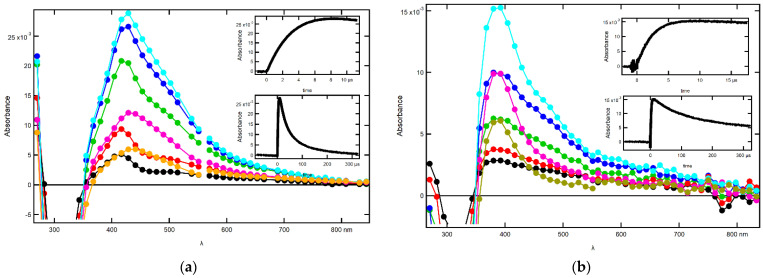
Transient absorption spectra recorded in N_2_O-saturated unbuffered aqueous solutions containing 0.05 mM of 2-SeU (**a**) at pH 4, 500 (●), 1 μs (●), 3 μs (●), 5 μs (●), 8 μs (●), 50 μs (●), and 100 μs (●) after electron pulse. Insets: time profiles representing growth (upper) and decay (bottom) of transient absorption at λ = 440 nm; (**b**) at pH = 10, 300 ns (●), 500 ns (●) 1 μs (●), 2 μs (●), 10 μs (●), 100 μs (●), and 300 μs (●) after electron pulse. Insets: time profiles representing growth (upper) and decay (bottom) of transient absorption at λ = 390 nm.

The time evolution of absorption spectra observed after pulse irradiation of N_2_O-saturated aqueous solutions containing 0.05 mM 2-SeU at pH 4 yielded a slight change in spectra as far as the position of the absorption maximum is concerned. With the time elapsed from 500 ns to 8 μs, a weakly developed absorption band with a maximum at λ = 410 nm underwent further substantial growth (top inset in Figure 2a), and a simultaneous and gradual small shift of a maximum to λ = 440 nm (Figure 2a). With time further elapsed, the absorption band with λ_max_ = 440 nm started to decay (bottom inset in Figure 2a) and at longer times (50 μs and 100 μs), after the pulse still dominated the spectra.

The subsequent chemical system subjected to irradiation was the basic aqueous solution at pH = 10 containing the same concentration of 2-SeU (0.05 mM). The spectral changes observed for the low concentration of 2-SeU are different from those observed at pH = 4. First of all, the absorption spectra at short time domain up to 1 μs are characterized by a weakly pronounced maximum at λ = 390 nm (Figure 2b), which underwent further substantial growth (top inset in Figure 2b), without, however, a shift of a maximum. With the time further elapsed up to 10 μs, the absorption spectrum starts to reveal a shoulder within 440–500 nm range. An appearance of a shoulder finds its justification in spectral features observed in solutions at pH 10 containing a high concentration of 2-SeU (1 mM), showing an absorption band with λ_max_ = 440 nm (Figure S1b in Supplementary Materials), and indicates the presence of dimeric selenium radical anions, in low concentrations. The absorption band with λ_max_ = 390 nm started to decay (bottom inset in Figure 2b), and at longer times (100 μs and 300 μs), the pulse still dominated the spectra, without a shoulder, however (Figure 2b).

To summarize, the time evolution of spectral features observed in solutions containing a low concentration of 2-SeU at these two pHs tentatively indicates the dominant contribution of dimeric selenium radicals at pH 4 and presumably of ^●^OH adducts to the C5 = C6 double bond at pH 10. These observations are also in line with the values of equilibrium constants (*K*) measured at these pHs (vide infra in Section 2.1.4).

#### 2.1.3. Influence of pH on the Time Evolution of Absorption Spectra at High Concentrations of 2-SeU

The spectral changes observed after pulse irradiation of N_2_O-saturated solution containing 1 mM of 2-SeU at pH 4 yielded less complex series of spectral changes in comparison to the solution containing 0.05 mM of 2-SeU. Only at 100 ns after electron pulse, a weakly developed absorption band with a maximum at λ = 410 nm, similar to that recorded for a low concentration of 2-SeU, was observed. However, with the time elapsed, this absorption band underwent a substantial growth (top inset in Figure S1a in Supplementary Materials) and a simultaneous shift of a maximum to λ = 440 nm, starting from 200 ns up to 1 μs after the electron pulse (Figure S1a in Supplementary Materials). With the time further elapsed, the absorption band with λ_max_ = 440 nm started to decay (bottom inset in Figure S1a in Supplementary Materials), and at longer times (50 μs and 100 μs), the pulse still dominated the spectra.

In contrast to solutions containing 0.05 mM of 2-SeU, the spectral changes observed for the highest concentration of 2-SeU (1 mM) at pH 10 are very similar to those observed at pH = 4 (Figure S1b in Supplementary Materials). The absorption spectra at short and long time domains are characterized by the absorption band with λ_max_ = 440 nm with similar growth and decay kinetic profiles (top and bottom insets in Figure S1b in Supplementary Materials).

To summarize, the time evolution of spectral features observed in solutions containing a high concentration of 2-SeU tentatively indicates a dominant contribution of dimeric selenium radicals and radical anions at pH 4 and pH 10, respectively.

#### 2.1.4. Equilibrium Constants and Rate Constants of Reactions Involved in Equilibrium

For both pHs, the maximum value of the 440 nm absorbance is dependent on the 2-SeU concentration. When this is increased from 0.05 mM to 1 mM, *G* × ε increases from 1.3 × 10^−3^ dm^3^ J^−1^ cm^−1^ to 4.3 × 10^−3^ dm^3^ J^−1^ cm^−1^ for pH = 4 (vide inset in Figure 1a) and from 0.5 × 10^−3^ dm^3^ J^−1^ cm^−1^ to 2.9 × 10^−3^ dm^3^ J^−1^ cm^−1^ for pH = 10 (vide inset in Figure 1b). This increase cannot be accounted for by an increase in ^●^OH concentration scavenged by 2-SeU due to the higher concentration of the latter [47]. Based on our earlier work with 2-TU [46], it rather points to the existence of an equilibrium, analogous to that in solutions containing 2-TU, where dimeric selenium radicals or radical anions are formed, which are responsible for the strong absorption at λ_max_ = 440 nm.

The equilibrium constant *K* can be obtained from Equation (1), where *A*_0_ is the absorbance at λ_max_ = 440 nm in 2-SeU solutions of 1 mM and *A* is the absorbance at λ_max_ = 440 nm at a given concentration of 2-SeU.

*A*_0_/*A* − 1 = K^−1^ [2-SeU]^−1^ (2)

**Figure 3:**
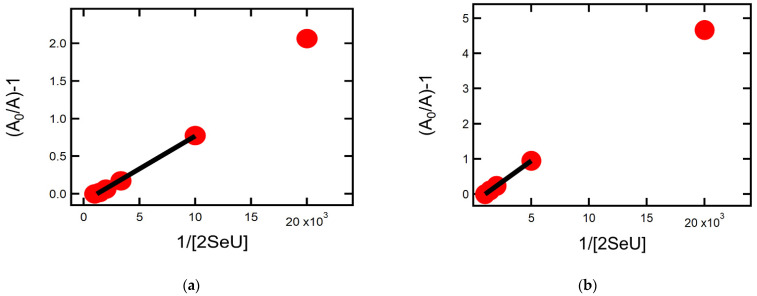
Dependence of the term *A*_0_/*A* − 1 at λ = 440 nm on the reciprocal of 2-SeU concentration in the pulse radiolysis of N_2_O-saturated unbuffered aqueous solutions at pH = 4 (**a**) and pH 10 (**b**).

In Figure 3, the term *A_0_*/*A* − 1 is plotted against the reciprocal of 2-SeU concentration for pH 4 (panel a) and pH 10 (panel b), respectively. From the reciprocal values of the slopes of these linear plots, *K* values 13.1 × 10^3^ M^−1^ and 4.4 × 10^3^ M^−1^ were obtained for pH 4 and pH = 10, respectively (Table 1). Interestingly, these *K*-values are significantly different for 2-SeU as opposed to 2-TU, where the *K*-values of the analogous equilibrium for pH 4 and 10 were very close to each other [46].

Moreover, the *K*-value determined for 2-SeU at pH 4 is ~three-fold higher than the *K*-value determined in the analogous pH condition for 2-TU. On the other hand, the *K*-value determined for 2-SeU at pH 10 is nearly the same as the *K*-value determined in the analogous pH condition for 2-TU.

**Figure 4:**
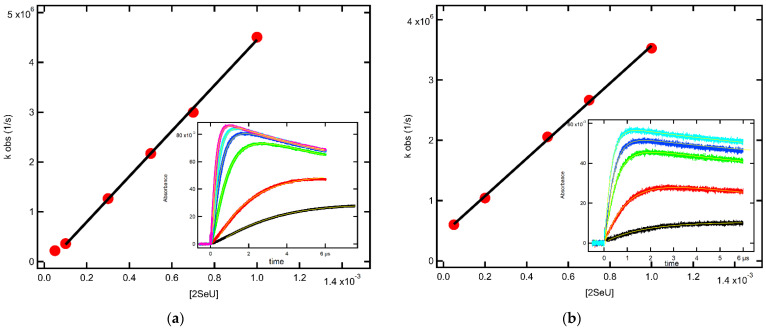
(**a**) Plots of the observed pseudo-first-order rate constants of the formation of the 440 nm absorption as a function of 2-SeU concentration in N_2_O-saturated unbuffered aqueous solutions at pH = 4. Inset: Time profiles of transient absorption at λ = 440 nm at various concentration of 2-SeU: from the bottom 0.05 mM (**−**), 0.1 mM (**−**), 0.3 mM (**−**), 0.5 mM (**−**), 0.7 mM (**−**), and 1 mM (**−**); (**b**) plots of the observed pseudo-first-order rate constants of the formation of the 440 nm absorption as a function of 2-SeU concentration in N_2_O-saturated unbuffered aqueous solutions at pH = 10. Inset: Time profiles of transient absorption at λ = 440 nm at various concentration of 2-SeU: from the bottom 0.05 mM (**−**), 0.2 mM (**−**), 0.5 mM (**−**), 0.7 mM (**−**), and 1 mM (**−**).

Kinetic treatment of the equilibration process can also be represented by Equation (2), where *k*_obs_ is the experimental pseudo-first-order rate constant for the formation of dimeric selenium radicals and radical anions (vide insets in Figure 4) at pH 4 and 10, respectively:

*k*_obs_ = *k*_forward_ [2-SeU] + *k*_backward_ (3)

This approach allows the determination of not only the equilibrium constant (*K*) but also the rate constants involved in the equilibrium, *k*_forward_ and *k*_backward_. Figure 4 shows plots based on Equation (2) for pH 4 (panel a) and pH 10 (panel b). Considering that such plots are characterized by high uncertainties in the intercepts, we decided to determine only *k*_forward_ from the slopes of the linear plots, which are equal to 4.6 × 10^9^ M^−1^s^−1^ and 3.1 × 10^9^ M^−1^s^−1^ for pH 4 and 10, respectively (Table 1). From the independent measurements of *K*-values (vide supra) and taking into account that *K* = *k*_forward/_*k*_backward_, *k*_backward_ were calculated as equal to 3.6 × 10^5^ s^−1^ and 7.0 × 10^5^ s^−1^ for pH 4 and 10, respectively. The values of *k*_forward_ (lower) and *k*_backward_ (higher) explain the lower stability of dimeric selenium radical anions at pH 10 in comparison to dimeric selenium radicals at pH 4.

#### 2.1.5. Oxidation of 2-SeU by ^●^OH Radicals at pH 4: Time-Resolved Conductivity

Time-resolved conductivity detection was successfully applied in the oxidation of 2-TU by ^●^OH radicals in order to resolve the problem of whether 2-TU oxidation proceeds via the formation of the monomeric sulfur radical cation or by separated coupled electron–proton transfer leading to dimeric sulfur radical [46]. Since an analogous dimeric radical is postulated for 2-SeU, we wanted to determine the nature of both the dimeric selenium radical and its precursor.

**Figure 5:**
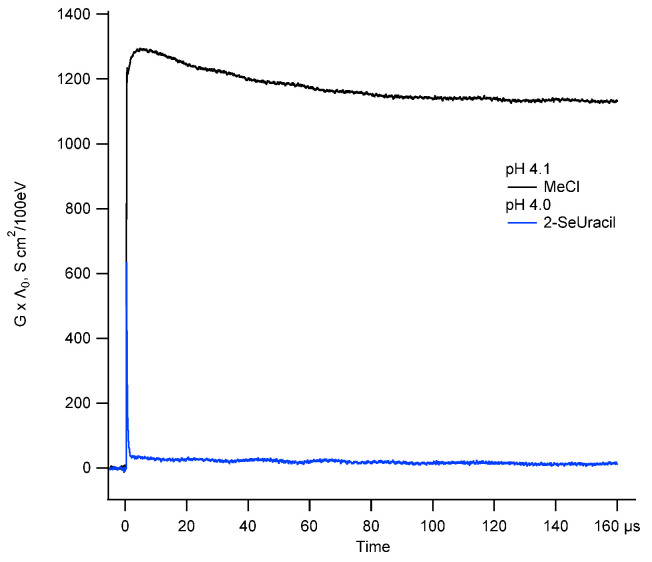
Comparison of equivalent transient conductivity changes represented as G × Λ_0_ vs. time after the pulse of electrons in N_2_O-saturated solutions containing 1 mM of 2-SeU at 4.0 (blue line) to CH_3_Cl-saturated aqueous solutions at the pH 4.1 (black line).

The results of our studies are shown in Figure 5. An instantaneous growth of conductivity followed by its fast decrease almost to the level recorded prior to the pulse (Figure 5, blue line) was observed after electron pulse in N_2_O-saturated solutions containing 1 mM of 2-SeU at pH = 4. The initial transient conductivity spike is a result of the net increase in conductivity due to the formation of hydrated electrons (e^−^_aq_) and protons (H^+^) during the radiolysis of water. N_2_O-saturated aqueous solutions e^−^_aq_ are quickly converted into ^●^OH radicals with the side product of hydroxide anions (HO^−^) within a few nanoseconds after the electron pulse. The following fast decrease of conductivity observed within 1 μs after the pulse is due to a stoichiometric neutralization reaction (H^+^ + HO^−^ → H_2_O) with *k* = 1.4 × 10^11^ M^−1^ s^−1^ [48] of highly conducting H^+^ by HO^−^. Upon completion of this reaction, a stable conductivity level of ~30 S cm^2^/100 eV is reached and remains unchanged for the next 160 μs. This picture of the conductance changes is exactly the same as that observed in N_2_O-saturated aqueous solutions containing 2-TU at pH 4.1 [46]; however, it is different from that observed in N_2_O-saturated aqueous solutions containing thiourea at pH 3.5 [20].

### 2.2. Oxidation of 2-SeU by ^●^N_3_ Radicals

#### 2.2.1. Influence of 2-SeU Concentration and pH on Absorption Spectra

Recently, it was shown that 2-SeU undergoes much easier oxidation than 2-TU [42]. In our earlier work, we found that 2-TU is oxidized by a relatively mild one-electron oxidant, the azide radical (^●^N_3_), which is one of the most important one-electron oxidants used extensively in radiation chemistry studies involving molecules of biological significance. The value of the standard reduction potential of the N_3_^●^/N_3_^−^ redox couple (E^0^) is equal to = 1.33 ± 0.01 V vs. NHE) [49]. Therefore, one would reasonably expect that the ^●^N_3_ radical can also easily oxidize 2-SeU.

**Figure 6:**
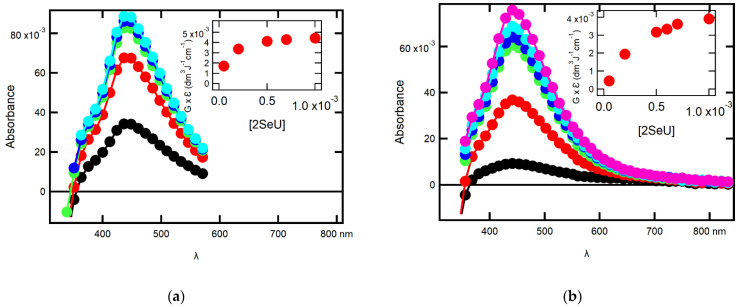
Transient absorption spectra recorded in N_2_O-saturated unbuffered aqueous solutions containing 30 mM NaN_3_: (**a**) at pH 6 containing 0.05 mM (●), 0.2 mM (●), 0.5 mM (●), 0.7 mM (●), and 1.0 mM (●) of 2-SeU recorded 10 μs, 6 μs, 3 μs, 2 μs, and 1.5 μs, respectively, after electron pulse; (**b**) at pH 10 containing 0.1 mM (●), 0.2 mM (●), 0.5 mM (●), 0.6 mM (●), 0.7 mM (●), and 1 mM (●) of 2-SeU recorded 8 μs, 3 μs, 2 μs, 1.7 μs, 1.5 μs, and 1.2 μs, respectively after electron pulse. Insets: Maximum absorbances (expressed as G × ε) recorded at λ = 440 nm in a function of 2-SeU concentration.

The chemical systems subjected to irradiation were the N_2_O-saturated aqueous solutions containing 30 mM NaN_3_ and 2-SeU in the concentration range of 0.05 mM to 1 mM at pH 6 and in the concentration range of 0.1 mM to 1 mM of 2-SeU at pH 10. In these solutions, ^●^OH radicals formed during the radiolysis of water quantitatively oxidize azide anions (N_3_^−^) to azide radicals (N_3_^●^). The spectral changes observed in these solutions are presented in Figure 6. The recording times are selected at the maximum of the absorbance measured at λ = 440 nm after electron pulse for the specified concentration of 2-SeU. It is clearly seen that the absorption spectra recorded at pH 6 for the concentration range 0.5 mM–1 mM of 2-SeU are dominated by the absorption band with λ_max_ = 440 nm, with nearly the same intensities expressed as G × ε_440_ (Figure 6a, inset). For the remaining two lower concentrations of 2-SeU (0.05 mM and 0.1 mM), the intensities of absorption bands are substantially lower (Figure 6a, inset); however, their shapes and positions of the absorption maxima are exactly the same as the absorption spectra recorded for the higher concentrations (Figure 6a). The absorption spectra recorded at pH 10 for the concentration range 0.1 mM to 1 mM of 2-SeU are dominated by the absorption band with λ_max_ = 440 nm, with the continuous growth of the intensity expressed as G × ε_440_ with a concentration of 2-SeU (Figure 6b, inset). However, it has to be noted that the intensities of the absorption bands with λ_max_ = 440 nm at the highest concentration of 2-SeU are slightly different. The intensity of the absorption band recorded in solutions at pH 6 is higher by more than 10% in comparison to pH 10.

It has to be noted that the intensities of absorptions bands with λ_max_ = 440 nm were recorded at the highest concentration of 2-SeU at both pHs in solutions where the ^●^N_3_ radical was an oxidant are higher (Figure S2 in Supplementary Materials) than those recorded in solutions where HO^●^ was an oxidant (Figure S1 in Supplementary Materials).

#### 2.2.2. Influence of pH on the Time Evolution of Absorption Spectra at Low Concentrations of 2-SeU

**Figure 7:**
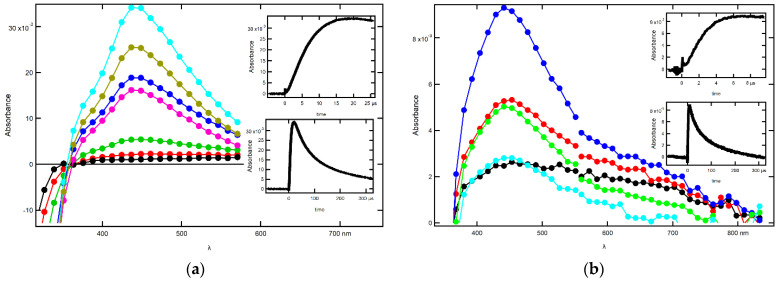
Transient absorption spectra recorded in N_2_O-saturated unbuffered aqueous solutions containing 30 mM of NaN_3_ and 0.05 mM of 2-SeU: (**a**) at pH = 6, 500 ns (●), 1 μs (●), 2 μs (●), 6 μs (●), 18 μs (●), 50 μs (●), and 100 μs (●) after electron pulse; (**b**) at pH 10, 1 μs (●), 2 μs (●), 7 μs (●), 50 μs (●), and 100 μs (●) after electron pulse. Insets: time profiles representing growth (upper) and decay (bottom) of transient absorption at λ = 440 nm.

Transient absorption spectra recorded at 500 ns and 1 μs after the electron pulse in N_2_O-saturated aqueous solutions containing 30 mM of NaN_3_ and 0.05 mM 2-SeU at pH 6 are characterized by a very weak and flat absorption band in the region 350–600 nm, which starts to develop and at 2 μs after the electron pulse is characterized by the absorption maximum with λ = 440 nm (Figure 7a). This picture resembles the spectral changes in the same wavelength region in an analogous solution containing 2-TU. However, contrary to 2-TU solutions, a narrow and distinct absorption with λ_max_ = 320 nm was not observed. Due to the stronger absorption of 2-SeU in the ground state (Figure S3 in Supplementary Materials), measurements for λ < 340 nm were not possible. With time further elapsed, a gradual increase in the intensity of the absorption band with λ_max_ = 440 nm up to 18 μs (top inset in Figure 7a) was observed. At longer times, this absorption band started to decay (bottom inset in Figure 7a); however, it still dominates the absorption spectrum at 50 μs and 100 μs after the pulse. This picture is different from that observed in 2-TU solutions, where the absorption band assigned to dimeric sulfur radical did not develop at all. These observations can be rationalized by differences in equilibrium constants (*K*) for the respective dimeric radicals.

The subsequent chemical system subjected to irradiation was the basic aqueous solution at pH = 10 containing the same concentration of NaN_3_ and 2-SeU (0.05 mM). The spectral changes observed for the low concentration of 2-SeU are very similar to those observed at pH = 6 (Figure 7b). The only difference concerns the intensity of the absorption band at λ_max_ = 440 nm, which is four-fold lower in comparison to pH 6.

To summarize, the time evolution of spectral features observed in solutions containing a low concentration of 2-SeU tentatively indicate a dominant contribution of dimeric selenium radicals and dimeric radical anions at pH 6 and pH 10, respectively.

#### 2.2.3. Influence of pH on the Time Evolution of Absorption Spectra at High Concentrations of 2-SeU

The spectral changes observed after pulse irradiation of N_2_O-saturated solution containing 30 mM of NaN_3_ and 1 mM of 2-SeU at pH 6 and 10 yielded similar spectral changes (Figure S2 in Supplementary Materials). With the time elapsed from 100 ns to −1.5 μs, a weakly developed absorption band with a maximum at λ = 440 nm underwent a substantial growth (top insets in Figure S2 in Supplementary Materials). With the time further elapsed, the absorption band with λ_max_ = 440 nm started to decay (bottom insets in Figure S2 in Supplementary Materials), and at longer times (50 μs and 100 μs), the pulse still dominated the spectra. The only difference concerns the intensity of the absorption band at λ_max_ = 440 nm, which is ~15% lower in comparison to pH 6 (Figure S2 in Supplementary Materials).

#### 2.2.4. Equilibrium Constants and Rate Constants of Reactions Involved in Equilibrium

As in the solutions where ^●^OH was an oxidant, the maximum value of the 440 nm absorbance is dependent on the 2-SeU concentration at both pHs. When this is increased from 0.05 mM to 1 mM, *G* × ε increases from 1.7 × 10^−3^ dm^3^ J^−1^ cm^−1^ to 4.4 × 10^−3^ dm^3^ J^−1^ cm^−1^ for pH = 6 (vide inset in Figure 6a) and from 0.1 mM to 1 mM, *G* × ε increases from 0.5 × 10^−3^ dm^3^ J^−1^ cm^−1^ to 4.0 × 10^−3^ dm^3^ J^−1^ cm^−1^ for pH = 10 (vide inset in Figure 1b). Again, this increase cannot be accounted for by an increase in ^●^N_3_ concentration scavenged by 2-SeU due to the higher concentration of the latter. It rather points to the existence of an equilibrium, analogous to that in solutions where HO^●^ was an oxidant, where dimeric selenium radicals or radical anions are formed, which are responsible for the strong absorption at λ_max_ = 440 nm.

The equilibrium constant *K* can also be obtained from Equation (1), where *A_0_* is the absorbance at λ_max_ = 440 nm in 2-SeU solutions of 1 mM and *A* is the absorbance at λ_max_ = 440 nm at a given concentration of 2-SeU.

**Figure 8:**
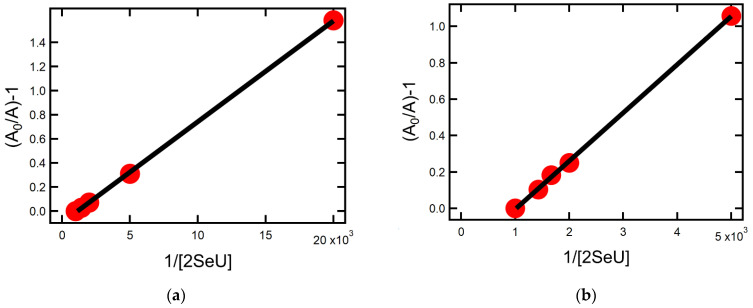
Dependence of the term *A*_0_/*A* − 1 at λ = 440 nm on the reciprocal of 2-SeU concentration in the pulse radiolysis of N_2_O-saturated unbuffered aqueous solutions containing 30 mM of NaN_3_ at pH = 6 (**a**), and pH 10 (**b**).

In Figure 8, the term *A*_0_/*A* − 1 is plotted against the reciprocal of 2-SeU concentration for pH 6 (panel a) and pH 10 (panel b), respectively. From the reciprocal values of the slopes of these linear plots, *K* values 11.9 × 10^3^ M^−1^ and 3.9 × 10^3^ M^−1^ were obtained for pH 6 and pH = 10, respectively (Table 1). These *K* values are nearly equal to those obtained where HO^●^ was an oxidant.

**Figure 9:**
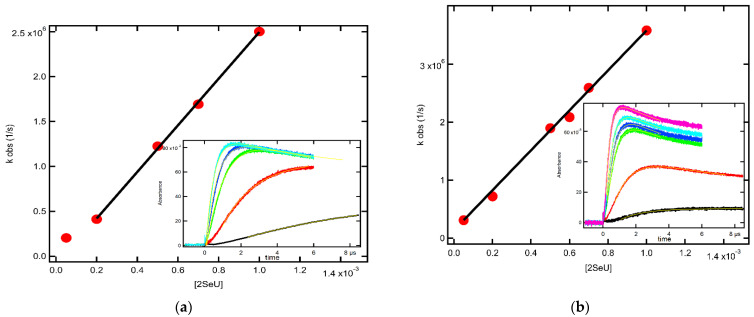
(**a**) Plots of the observed pseudo-first-order rate constants of the formation of the 440 nm absorption as a function of 2-SeU concentration in N_2_O-saturated unbuffered aqueous solutions at pH = 6 containing 30 mM NaN_3_. Inset: time profiles of transient absorption at λ = 440 nm at various concentration of 2-SeU: from the bottom, 0.05 mM (**−**), 0.2 mM (**−**), 0.5 mM (**−**), 0.7 mM (**−**), and 1 mM (**−**); (**b**) plots of the observed pseudo-first-order rate constants of the formation of the 440 nm absorption as a function of 2-SeU concentration in N_2_O-saturated unbuffered aqueous solutions at pH = 10 containing 30 mM NaN_3_. Inset: time profiles of transient absorption at λ = 440 nm at various concentration of 2-SeU: from the bottom 0.1 mM (**−**), 0.2 mM (**−**), 0.5 mM (**−**), 0.6 mM (**−**), 0.7 mM (**−**), and 1 mM (**−**).

Kinetic treatment of the equilibration process can also be represented by Equation (2), where *k*_obs_ is the experimental pseudo-first-order rate constant for the formation of dimeric selenium radicals/radical anions (vide insets in Figure 9) at pH 6 and 10, respectively. Figure 9 shows plots based on Equation (2) for pH 6 (panel a) and pH 10 (panel b). Considering that such plots are again characterized by high uncertainties in the intercepts, we only determined *k*_forward_ from the slopes of the linear plots equal to 2.6 × 10^9^ M^−1^s^−1^ and 3.7 × 10^9^ M^−1^s^−1^ for pH 6 and 10, respectively (Table 1). Using the same approach as above (vide Section 2.1.4), *k*_backward_ were calculated equal to 2.2 × 10^5^ s^−1^ and 9.5 × 10^5^ s^−1^ for pH 6 and 10, respectively.

### 2.3. Theoretical Calculations

#### 2.3.1. Neutral and Deprotonated Forms of 2-SeU

**Figure 10:**
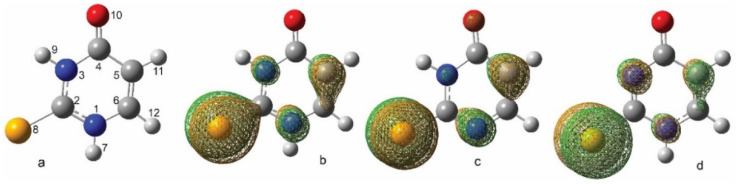
Optimized geometry of neutral and deprotonated 2-SeU in solution phase (**a**,**c**,**d**). HOMO of (**b**) neutral 2-SeU, (**c**) N_1_ deprotonated, and (**d**) N_3_ deprotonated 2-SeU (isocontour value = 0.04 a.u.).

Optimized geometries of neutral and singly deprotonated forms of 2-SeU in the solution phase are presented in Figure 10, along with their highest occupied molecular orbitals (HOMO). Substitution of S atom by Se atom in 2-thiouracil slightly decreases distances between the atoms in the molecular ring (< 1%). Tautomerization relocating hydrogen atoms from nitrogen atoms to either oxygen or selenium atoms is endothermic by 10 or 11 kcal mol^−1^, respectively. Therefore, the contribution of tautomeric forms seems negligible in the studied mechanism in aqueous solutions at room temperatures, and free-radical-induced transformations of these structures are not being considered further. The highest electronic density in neutral 2-SeU is localized, analogously to 2-thiouracil [46,50], on chalcogen heteroatom, Se8, and C5, as well as, in lower degrees, on atoms C6, N1, and N3. Deprotonation of 2-SeU does not affect the overall pattern of electronic density distribution in the molecule but slightly decreases the electronic density on C6 and N3, or only at C6, in forms of the molecule deprotonated at N1 or N3, respectively (Figure 10). All of the lobes of HOMO orbitals are perpendicular to the plane of the molecule analogous to 2-TU.

Upon encounter with the 2-SeU molecule, electrophilic ^●^OH mostly targets positions with the highest electronic densities, and in effect, the formation of monomeric intermediates comparable to the ones observed in its sulfur analog can be expected [46]. We have shown that among routes of oxidation by ^●^OH in 2-TU, the most prominent is proton-coupled electron transfer, PCET (net resulting in H abstraction at either N1 or N3 atoms), and ^●^OH addition to the most electrophilic ring positions (at C5 or C6 atoms). Ionization potential, IP, of 2-SeU is 5.98 eV, which is 0.52 eV lower than in 2-TU, and comparable to thiourea at 6.07 eV (all computed in solution phase). This may suggest that 2-SeU would undergo, like thioureas [20], direct one-electron oxidation to radical cation. In 2-TU, we did not find evidence of 2TU^●+^ (**1^●+^**) radical cation formation at pH ≥ 4. This could have been related to the higher IP in 2-TU or to the different positioning of lobes of HOMO orbitals, which in thioureas are in the plane of the molecule. Either deprotonated form of 2-SeU can donate electrons easier than a neutral molecule because its electron affinity is ~0.8 eV lower than the IP of the neutral molecule. Electron affinity computed in the solution phase for molecule deprotonated at N1 in form **1_a_^−^** or deprotonated at N3 in the form of **1_b_^−^** is 5.18 and 5.15 eV, respectively.

#### 2.3.2. Monomeric Intermediates Generated in Neutral and Deprotonated Forms of 2-SeU

Optimized geometries of all monomeric intermediates generated upon one-electron oxidation of 2-SeU by OH radicals, PCET, and ^●^OH addition to neutral and deprotonated forms of the molecule are compiled in Supplementary Materials (see Figure S4 and Figure S5 in Supplementary Materials) along with their thermochemistry values (Table S1 in Supplementary Materials).

**Figure 11:**
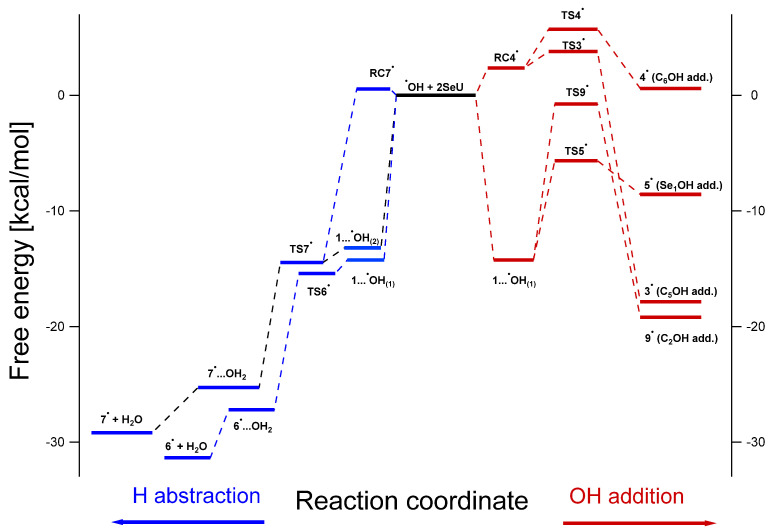
Relative energy profiles for the H abstraction (blue) and OH addition (red) from/to neutral 2-SeU, respectively, computed at ωB97x/aug-cc-pvtz-pp level of theory in aqueous phase (PCM).

Showing many structural similarities with 2-TU, oxidation of 2-SeU by ^●^OH radicals is expected to proceed like in 2-TU via two main routes: H-atom abstraction and ^●^OH-addition. Figure 11 illustrates computed (at ωB97x/aug-cc-pvtz-pp level of theory in aqueous phase) relative energy profiles for both processes in neutral 2-SeU.

*Selenyl Radicals*. H abstraction route is barrier-less, like in 2-TU, and leads to the formation of selenyl (equivalent of thiyl) radicals 6^●^ and 7^●^ (see optimized geometries and spin densities in Figure S4 in Supplementary Materials). Like in the sulfur analog, the first step of H-atom abstraction proceeds through the barrierless formation of two-center, three-electron (2c-3e) ^●^OH adducts to Se atom assigned as 1…^●^OH_(1)_ and 1…^●^OH_(2)_, when OH radical approaches selenium from the side of N1 and N3, respectively. This reaction step is over 7 kcal mol^−1^ more preferable thermodynamically than the formation of analogous sulfuric intermediates (vide Table S2 in Supplementary Materials). Unlike in 2-TU, there seems to be no additional path for the formation of radical 7^●^ via direct abstraction of the H atom at the N3 position when ^●^OH radical approaches this position from the O10 side of the ring, as we could not find an analogous transition state (TS7_2_^●^) obtained in earlier studies in the sulfur analog [46]. Therefore, we assumed TS7^●^ to be an alternative transition state for this particular route of ^●^OH radical attack at the N3 position. This route has a small barrier of ~0.55 kcal mol^−1^ related to the formation of reaction complex RC7^●^.

*^●^OH Addition to C5 = C6 Double Bond*. ^●^OH addition to 2-SeU proceeds mostly at C5 and C6, giving radicals 3^●^ and 4^●^, respectively. The most stable rotamers of these OH adducts have H atoms pointing either away or towards the molecular ring in radicals 3^●^ or 4^●^, respectively. Transition state TS3^●^ has a lower barrier energetically than TS4^●^, suggesting regioselectivity of ^●^OH addition at position C5, analogously to results of computations performed for 2-TU. Intrinsic Reaction Coordinate (IRC) from either of the transition states leads to a common reactive complex RC^●^, i.e., a precursor formed upon encounter of ^●^OH with 2-SeU at the C5 = C6 double bond site. Illustration of respective energy levels of ^●^OH addition routes at positions C5 and C6 are summarized in Figure 11, and numerical values for given elementary reactions are summarized in Table S2 in Supplementary Materials. Interestingly, in 2-TU, radical 4^●^ was more thermodynamically stable than radical 3^●^. In selenium, uracil analog radical 4^●^ formation has a higher barrier, and its formation is actually endothermic by 0.59 kcal mol^−1^. Considering lower stability and higher barrier of formation of 4^●^, we can assume that its yield should be insignificant.

*^●^OH Addition to C2 = S8 Double Bond*. Considering the variety of ^●^OH addition pathways, we analyzed structures and barriers of ^●^OH addition at positions C2 and Se8. Both of these ^●^OH adducts are thermodynamically more stable than 4^●^ and can exist in two rotameric forms of very close respective energies. The paths of their formation, however, start from 2c-3e ^●^OH adducts to Se (1…^●^OH_(1)_, 1…^●^OH_(2)_) and need to overcome large energy barriers at transition states TS5^●^ and TS9^●^. Considering that H-atom abstraction at N1 and N3 proceeds from the same 2c-3e ^●^OH adducts to Se8, we can safely assume that yields of these ^●^OH adducts should be negligible. However, if vacuum IRC is providing misleading guidance and the solution phase reaction pathway reaching TS5^●^ and TS9^●^ can be realized from different starting complexes, this would grant a lower barrier for producing OH adducts 5^●^ and 9^●^, making them more relevant in the following discussion.

Upon ^●^OH’s encounter with either of singly deprotonated forms of 2-SeU, **1_a_^−^** and **1_b_^−^** (subscripts ‘a’ or ‘b’ indicate the site of deprotonation at N1 or N3 atoms, respectively), the same two possible routes of reactivity can be observed as in the neutral form of the molecule. Deprotonated monomer-type transients (1_a_^−^…^●^OH_(1)_, 1_a_^−^…^●^OH_(2)_, 1_b_^−^…^●^OH_(1)_, 1_b_^−^…^●^OH_(2)_, 3_a_^●−^, 4_a_^●−^,3_b_^●−^, 4_b_^●−^, 10^●−^) optimized geometries (along with their spin densities) produced in either H atom abstraction or OH addition from/to anionic forms of 2-SeU are compiled in Figure S5 (vide Supplementary Materials). Illustration of relative energy profiles for possible routes of ^●^OH reactivity with anionic forms of 2-SeU are presented in Figure S8A–D (vide Supplementary Materials). Free energies of all elementary reactions are compiled in Table S2 (vide Supplementary Materials). In both deprotonated forms, the most preferable thermodynamically route leads to the formation of selenium analog of thiyl radical anion 10^●−^. Analogous observation has been performed previously for anionic forms of 2-TU. In deprotonated 2-SeU, H atom abstraction via PCET (or other electron reaction) is more favorable by more than in neutral 2-SeU by an estimated 0.8 eV difference in IPs mentioned earlier. It is worth noting that, unlike in the neutral form, in a singly deprotonated selenobase, a PCET process can only proceed on one side of a selenium atom, where a nitrogen atom is protonated, whereas the opposite side of the selenium atom would accommodate a 2c-3e ^●^OH adduct, which cannot undergo PCET and presumably have a longer lifetime due to hydrogen bonding between H atom (of OH) and deprotonated nitrogen site (for structures, see Figure S5 in Supplementary Materials). Since 1_a_^−^…^●^OH_(2)_ and 1_b_^−^…^●^OH_(1)_ are involved in a PCET, they would disappear instantaneously, producing radical anion 10^●~^. However, 1_b_^−^…^●^OH_(2)_ and 1_a_^−^…^●^OH_(1)_, not having barrier less PCET channel of reactivity, should be detectable somehow longer than any hemi-bonded OH adducts involved in PCET (either in neutral or anionic form of 2-SeU). In both of these hydrogen-bonded 2c-3e OH adducts, the hemibond can either dissociate back to the original selenouracil anion and ^●^OH radical, or it can dissociate with electron transfer, producing selenyl radical 6^●^/7^●^ and OH^−^. The latter path is energetically favorable over backward reactions by 0.7 or 1.3 kcal/mol, respectively (Figure S8D). Even though these OH-adduct mediated electron transfers (sometimes categorized as inner-sphere electron transfers) are preferable over backward reactions, they still have substantial barriers of 9.9 and 9.8 kcal/mol. The estimated reaction barrier in the dissociation of NCSeOH^●−^ into NCSe^●^ and OH^−^ is about 18.9 kcal/mol, yet NCSeOH^●−^ cannot be observed at a pH higher than 13.5. Therefore, we can safely assume that we should not be able to observe 1_b_^−^…^●^OH_(2)_ and 1_a_^−^…^●^OH_(1)_ intermediates at nanosecond time scales.

Among OH-adducts to deprotonated 2-SeU again, most favorable is the formation of the OH adduct in positions C5, 3_a_^●−^ and 3_b_^●−^, from molecules deprotonated at N1 and N3, respectively (vide Figure S8B,C in Supplementary Materials).

#### 2.3.3. Dimeric Intermediates Generated in Neutral and Deprotonated Forms of 2-SeU

*2c-3e Se-Se Bonded Dimeric Radicals and Radical Anions.* At higher concentrations of 2-SeU, we expected, analogously to 2-TU, the formation of a variety of 2c-3e dimer radicals. At very low pH, the existence of radical cation 1^●+^ can be anticipated. This radical cation can dimerize with a neutral parent molecule, producing 2c-3e Se-Se bonded dimer radical cation 2^●+^, which structurally resembles sulfur analog postulated at low pH in aqueous solutions of 2-TU [46]. Dimeric radical cation 2^●+^ can exist in three different isomeric forms produced by 180° rotation of rings around either or both of C–Se bonds. The variation of energy between all of them is less than 0.34 kcal mol^−1^; hence, all can be observed at room temperature. The optimized geometry of the isomer with the lowest energy has a structure analogous to its sulfur analog [46] with two ring planes positioned at a −99° angle and with oxygen atoms pointing outwards of the center of the molecule (Figure S6 in Supplementary Materials).

Analogously to 1^●+^, its deprotonated forms of thiyl-like radicals 6^●^/7^●^ can also dimerize with neutral 2-SeU, producing neutral 2c-3e Se-Se bonded dimer radical 2^●^. This neutral dimer can coexist in a variety of isomeric forms depending on whether radical 6^●^ or 7^●^ undergoes dimerization, and depending on what kind of stabilizing hydrogen bonding occurs between two nitrogen sites on both dimerizing molecules. The geometry of the most stable structure of the neutral dimer 2^●^ is presented in Figure S6 (vide Supplementary Materials). In comparison to dimer 2^●+^, the arrangement of two binding fragments in radical 2^●^ is spatially different but seems analogous to the structure of neutral dimers previously postulated for its sulfur analog [46]. This isomer is thermodynamically 3.7 kcal mol^−1^ less stable than dimeric radical cation 2^●+^ discussed earlier, yet it is about 3.8 kcal mol^−1^ more stable than its sulfur analog, whose formation was exothermic by only 0.55 kcal mol^−1^ [46]. The energy variation between 2^●^ isomers is about 0.6 kcal mol^−1^. Therefore, it is conceivable to believe that all these isomers can be observed at room temperature. Based on the higher formation energy of 2^●^ in 2-SeU, one can expect a sizably higher formation equilibrium constant and its overall higher stability than an analogous intermediate produced from 2-TU.

Dimerization of radicals 6^●^/7^●^ with deprotonated forms of 2-SeU (**1_a_^−^,1_b_^−^**), above its p*K*_a_, produces various isomeric forms of 2c-3e Se-Se bonded dimer radical anion 2^●−^. Its structure resembles neutral dimer radical 2^●^ and can be generated by site-specific deprotonation from isomers of radical 2^●^. The structure of the most thermodynamically stable isomer of dimer radical anion 2^●−^ is presented in Figure S6 (vide Supplementary Materials). This intermediate formed upon dimerization of radical 6^●^ with 1_a_^−^ anion has a formation energy of −8.7 kcal mol^−1^, which makes it most stable among all the dimers discussed so far and about 2.7 kcal mol^−1^ more stable than its sulfur analog [46]. Based on that, one can assume that 2^●−^ will also have a higher formation equilibrium constant than an analogous intermediate produced from 2-TU.

Dimerization of selenyl radical anion 10^●−^ readily formed in basic solutions with deprotonated forms of 2-SeU (**1_a_^−^,1_b_^−^**) should produce various forms of dimer radical dianion 2^●−2^. The most stable isomer formed on the encounter of **1_a_^−^** and 10^●−^ (vide structure Figure S6 in Supplementary Materials) or on deprotonation of 2^●−^ has, surprisingly, 3.5 kcal mol^−1^ formation energy, which makes it least stable among all the 2c-3e Se-Se dimers, yet still more stable than many neutral dimers produced from 2-TU (vide Table S3 in Supplementary Materials).

#### 2.3.4. UV-Vis Spectra of Monomeric and Dimeric Intermediates Derived from 2-SeU

**Figure 12:**
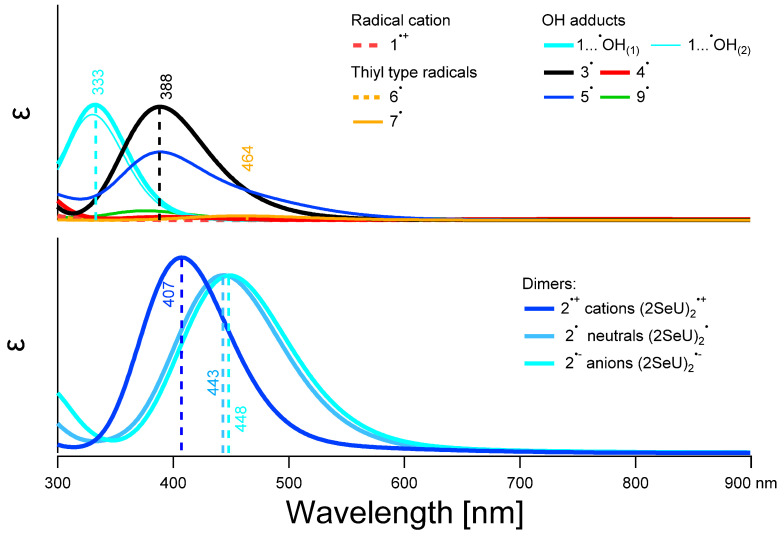
TD-DFT calculated absorption spectra of potential transients (see legend for symbols) produced in ^●^OH–induced oxidation of 2SeU in water at pH lower than its first p*K*_a_ (7.18).

Calculated UV-Vis spectra of monomer type transients derived from 2-SeU (1^•+^, its deprotonated forms of selenyl-like radicals 6^•^/7^•^, and various ^•^OH adducts) and a group of dimer radicals are shown in the upper and lower part of Figure 12, respectively.

Monomer-type transients derived from singly deprotonated 2-SeU along with neutral and anion dimer radical spectra are presented in Figure S7 (vide Supplementary Materials). Computed spectra extend from 300 nm up to 900 nm since, similarly to 2-TU derived intermediates, it was not possible to measure absorbance below 300 nm. Therefore, we are not reporting the results of calculations for 2-SeU below 300 nm. Dissimilarly to 2-TU, radical cation 1^●+^ and its deprotonated forms 6^●^ and 7^●^ derived from 2-SeU do not absorb light beyond 550 nm. Among all monomer-type transients derived from 2-SeU expected to be produced at pH below p*K*_a_ of 2-SeU and observable before dimerization, one can assume to see radical 3^●^ as well as very weak absorbance assigned to radicals 6^•^ and, in a lesser extent, 7^•^. These radicals have electronic transitions in the range where dimeric radical cations and/or dimeric radicals (2^●+^, 2^●^) have strong transitions as well (vide infra). Therefore, the only possibility to observe them would be at the lowest concentration of the solute when dimerization processes are kinetically limited. The most abundant at low concentrations, radical 6^●^, has strongest electronic transition at λ ~419 nm, which is 17-fold weaker and 212 nm blue-shifted from its sulfur analog [46].

Calculated electronic transitions for 2c-3e Se-Se bonded dimer radicals 2^●+^, 2^●^, 2^●−^ (Figure 12) qualitatively resemble their sulfur analogs observed in previous studies of 2-TU. Again, dimer radical cation absorbs slightly stronger (~9%) and has λ_max_ = 407 nm shifted blue from neutral and anionic analogs with λ_max_ = 443 nm and 448 nm, respectively. Individual isomers in a group of differently charged dimers do not differ much from their lowest energy forms and are not included for clarity. It is worthy to note that 2^●+^ is shifted blue, whereas neutral and anionic dimers (2^●^_,_ 2^●−^, and 2^●−2^) are shifted red in comparison to 2-TU derived analogs observed and described previously [46].

## 3. Discussion

### 3.1. Characterization of Radical Species: Spectral, Kinetic, and Energetic Parameters

*2c-3e Se-Se Bonded Dimeric Radicals and Radical Anions.* Our earlier studies on one-electron oxidation of 2-TU by ^●^OH and ^●^N_3_ radicals reported the formation of the dimeric radicals and radical anions of 2-TU with 2c-3e sulfur-sulfur bond characterized by an absorption band with λ_max_ = 430 nm. The intensity of this absorption band was dependent upon the 2-TU concentration) [46]. Since similar observations were made with 2-SeU (see Figure 1 and Figure 6), we conclude that the dimeric radicals (2^●^) and radical anions (2^●−^) with 2c-3e selenium-selenium bond are formed (vide Figure S6). However, the position of absorption maxima of the bands assigned to them (λ = 440 nm) is shifted slightly red. This observation was confirmed by theoretical calculations showing λ_max_ = 443 and 448 nm for 2^●^ and 2^●−^, respectively (Figure 12).

We also demonstrate the existence of an equilibrium, analogous to that in solutions containing 2-TU, where dimeric selenium radicals (2^●^) and radical anions (2^●−^) are in equilibrium with selenyl-type radicals 6^●^/7^●^ (vide Figure S4 in Supplementary Materials). Interestingly, the *K*-values determined for 2-SeU (vide Table 1) at pH 4 and pH 6 (lower than the first p*K*_a_ of 2-SeU) are ≈ three-fold higher than the *K*-value determined at pH 4 for 2-TU [46]. This observation is again in line with theoretical calculations showing higher formation energy of 2^●^ in 2-SeU by about 3.8 kcal mol^−1^ in comparison to analogous intermediate produced from 2-TU and, consequently, the higher equilibrium constant and its overall higher stability. The values of *k*_forward_ 4.6 × 10^9^ M^−1^ s^−1^ and *k*_backward_ = 3.6 × 10^5^ s^−1^ for 2-SeU (vide Table 1) in comparison to *k*_forward_ 3.6 × 10^9^ M^−1^ s^−1^ and *k*_backward_ = 7.8 × 10^5^ s^−1^ for 2-TU [46] explain the higher stability of dimeric selenium radicals at pH 4.

On the other hand, the *K*-value determined for 2-SeU (vide Table 1) at pH 10 (higher than the first p*K*_a_ of 2-SeU) is nearly the same as the *K*-value determined at pH 10 for 2-TU [46]. However, based on theoretical calculations, the species (2^●−^) is about 2.7 kcal mol^−1^ more stable than its sulfur analog. At the moment, we cannot explain this discrepancy between experiments and calculations.

*^●^OH Adducts to C5 = C6 and C2 = S8 Double Bond*. Taking into account the molecular structure of the most energetically favorable tautomer of 2-SeU (Figure 10) and plausible primary reactions of ^●^OH radicals, it was reasonable to assume a double C = S bond at S8 position and a double C = C bond at C5 and C6 positions as the possible primary sites of ^●^OH attack. These additions lead to a barrierless formation of 2c-3e ^●^OH adducts to Se atom labeled as 1…^●^OH_(1)_ and 1…^●^OH_(2)_ and the OH adducts at C5 and C6 atoms labeled as 3^●^ and 4^●^, respectively. The first addition reaction is more preferable thermodynamically by 7 kcal mol^−1^ than the formation of analogous sulfur intermediates in 2-TU (Table S2 in Supplementary Materials). In turn, the formation of 3^●^ and 4^●^ in 2-SeU is characterized by similar energy barriers as in 2-TU. Based on these calculations, one can expect a sizably higher efficiency of 1…^●^OH_(1)_ and 1…^●^OH_(2)_ formation in 2-SeU than in 2-TU. Consequently, this fact should also be reflected in the higher efficiency of 2^●^ formation and simultaneous lower efficiency of the 3^●^ and 4^●^ formations in 2-SeU than in 2-TU (vide infra).

In the case of 2-TU, a more efficient formation of 3^●^ was observed since its formation proceeded through a lower energy barrier in comparison to 4^●^, though the latter radical was thermodynamically more stable than the most stable conformer of 3^●^. However, there was no reason to exclude the contribution of 4^●^ at a low concentration of 2-TU to the observed spectra [46]. In 2-SeU, considering the lower stability of 4^●^ (its formation is endothermic) and the higher barrier of its formation, we can safely assume that its yield should be negligible. Though radical 3^●^ is characterized by the calculated intensive absorption band with the maximum located at λ = 388 nm, its presence seems to be very weakly manifested in the spectrum recorded at pH = 4 for the lowest concentration of 2-SeU at the time where the dimerization process is still kinetically limited (Figure 2a). On the other, at pH = 10 for the lowest concentration of 2-SeU, the absorption spectrum is dominated by a well-pronounced absorption band with λ_max_ = 390 nm, which can be reasonably assigned to radical 3^●−^. Similar observations and assignments were made for 2-TU [46].

### 3.2. Justification of the Reaction Pathway Involving Hemibonded ^●^OH Adducts to Selenium Atom: One-Electron Transfer vs. Proton-Coupled Electron Transfer

Since the ionization potential (IP) of 2-SeU is lower than that of 2-TU and comparable to IP of thiourea, one could expect that 2-SeU would undergo direct one-electron oxidation to the respective radical cation (2-SeU^●+^). Contrary to our expectations, a decrease in the net conductivity below zero was not observed, which would imply consumption of H^+^ through a neutralization reaction by HO^−^ (vide Section 2.1.5), which are released with simultaneous formation of radical cation 1^●+^. Therefore, the formation of 1^●+^ followed by the formation of 2^●+^ does not seem to be the reaction pathway during ^●^OH-induced oxidation of the neutral form of 2-SeU at pH 4. Based on our earlier conductivity experiments with 2-TU, we suggest the following scenario for 2-SeU. The ^●^OH radicals form the hemibonded adduct to selenium (1…^●^OH), which decays by separated coupled electron–proton transfer. The HO^−^ generated in the inner-sphere electron transfer that leads to radical cation 1^●+^ is neutralized in the concerted reaction by the proton released either from either N1 or N3 atoms, leading to the formation of 6^●^ and 7^●^ radicals, respectively. Therefore, their formation is not associated with any net change of conductivity. This observation was crucial in determining the nature of both dimeric radical and its direct precursor.

### 3.3. Mechanism of the ^●^OH- and ^●^N_3_-Induced Oxidation of 2-Selenouracil

**Scheme 1:**
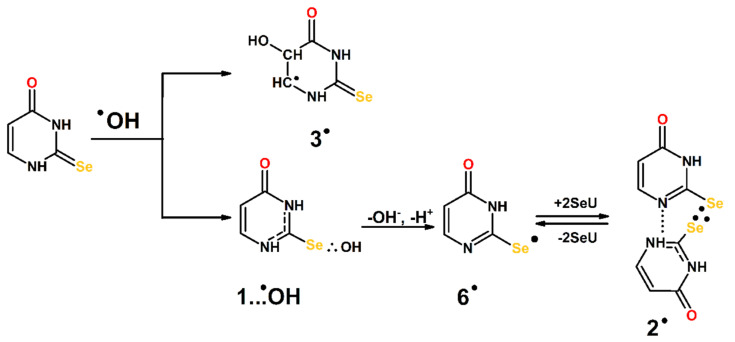
^●^OH-induced oxidation of 2-SeU at pH below its pK_a_ (●●●●● represents hydrogen bond in 2^●^ structure).

**Scheme 2:**
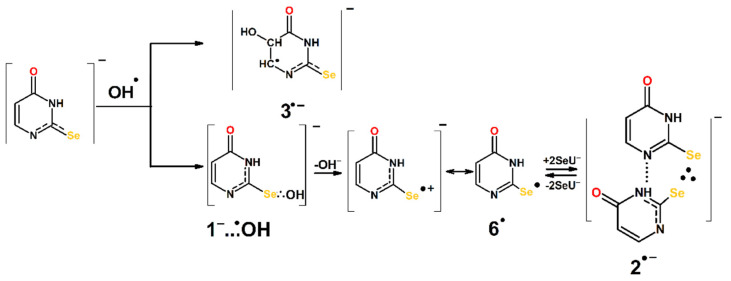
^●^OH-induced oxidation of 2-SeU at pH above its pK_a_ (●●●●● represents hydrogen bond in 2^●−^ structure).

**Scheme 3:**
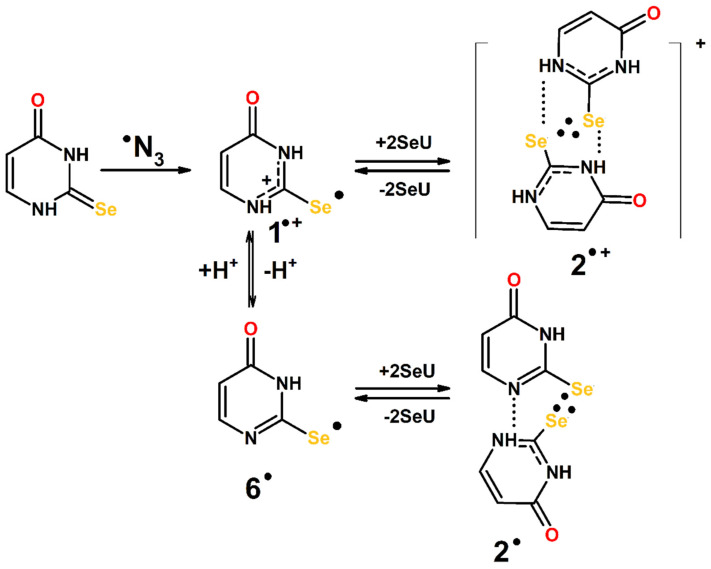
^●^N_3_-induced oxidation of 2-SeU at pH below its pK_a_ (●●●●● represents hydrogen bond in 2^●+^ and 2^●^ structures).

**Scheme 4:**
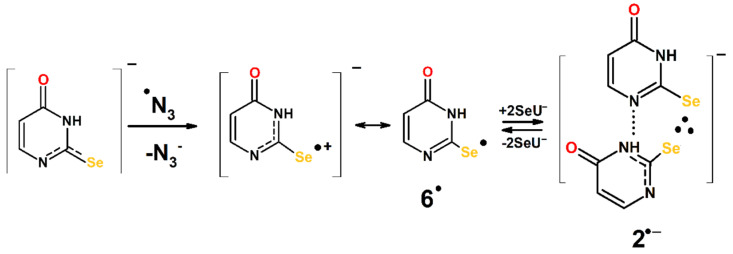
^●^N_3_-induced oxidation of 2-SeU at pH above its pK_a_ (●●●●● represents hydrogen bond in 2^●−^ structure).

Identification of transients and complementary theoretical calculations allow the formulation of the mechanisms for the ^●^OH and ^●^N_3_-induced oxidation of 2-SeU in aqueous solutions at pHs located below and above its pK_a_ (vide Scheme 1, Scheme 2, Scheme 3 and Scheme 4).

#### 3.3.1. Mechanism of the ^●^OH-Induced Oxidation of 2-Selenouracil

The initial steps are the additions of ^●^OH radical to the C2 = Se bond at the Se8 site and C5 = C6 bond at the C5 site, yielding a hemibonded adduct to selenium (1…^●^OH) and 3^●^ radical, respectively (Scheme 1).

The 1…^●^OH radical decays by separated coupled electron–proton transfer (vide Section 3.2), leading to the formation of 6^●^ and 7^●^ radicals. Since the formation of 6^●^ is thermodynamically preferred (slightly > 2 kcal mol^−1^) (vide Table S2 in Supplementary Materials), only this radical is presented for simplicity in the scheme. At a higher 2-SeU concentration, 6^●^ radicals are converted into dimeric radicals (2^●^); however, both radicals exist in equilibrium. It has to be stressed that this equilibrium at pH 4 is nearly totally shifted to the right for the highest concentration of 2-SeU used in our studies (vide inset in Figure 1a). The dimeric radical 2^●^ exists in neutral form since both substrates (6^●^ and 2-SeU) are present in neutral forms, too. Dissimilarly to 2-TU, the reaction pathway leading to 3^●^ radical is nearly negligible (vide supra).

Radicals 6^●^ and 7^●^, due to their very weak absorbance (substantially lower than their sulfur analogs), are not observed even at the lowest concentrations of 2-SeU, where equilibrium is not fully shifted to the right, and thus, they might be abundantly present. Moreover, these radicals absorb in the wavelength range where radicals 2^●^ have very strong absorptions (vide supra Section 2.3). In turn, 1…^●^OH radicals cannot be observed in our experiments since their absorption maxima are located in the region (Figure 12) of strong absorption of 2-SeU in the ground state (vide Figure S3 in Supplementary Materials).

At higher pH, located above the pK_a_ of 2-SeU, the initial steps of ^●^OH reactions with 2-SeU are practically the same and lead to the following transients 1^−^…^●^OH and 3^●−^ (Scheme 2).

The hemibonded adduct to sulfur (1^−^ …^●^OH) is then converted to radical 6^●^; however, unlike 1…^●^OH at pH 4, by inner-sphere electron transfer leading first to the radical cation with the location of the positive charge on the selenium atom. Its more significant resonance form is selenyl radical 6^●^. This is due to the well-known rule that neutral resonance structures are more important than charged resonance structures. Similarly, as for pH = 4, at a higher concentration of 2-SeU, radical 6^●^ is in equilibrium with the dimeric radical anion (2^●−^). The anionic character of dimeric radical is due to the fact that at pH = 10, 2-SeU exists in anionic form. Formation of 3^●−^ is also more preferable than the formation of 4^●−^. This is due to the higher thermodynamic stability and substantially lower energy barrier in comparison to 4^●−^ (vide supra).

#### 3.3.2. Mechanism of ^●^N_3_-Induced Oxidation of 2-Selenouracil

Since ^●^N_3_ radicals are commonly considered as one-electron oxidants, the initial step of 2-SeU oxidation at pH below its p*K*_a_ should lead to the respective radical cation (1^●+^) (Scheme 3). The formation of this radical cannot be observed directly because its absorption spectrum is located in the spectral range where 2-SeU absorbs very strongly (<300 nm) (vide Figure 12 and Figure S3 in Supplementary Materials).

In principle, the radical cation (1^●+^) could be a precursor of dimeric radical cation (2^●+^). However, its spectral feature is slightly different from that characterizing 2^●^ (Figure 12). According to calculated by us electronic transitions using the TD-DFT method, its absorption maximum is blue-shifted by ~30 nm, which should be clearly visible in the recorded absorption spectra. Since this is not the case (vide Figure 6 and Figure S2a in Supplementary Materials), one can assume another scenario. Radical cation (1^●+^) can undergo fast deprotonation to radical 6^●^, which further undergoes transformation to dimeric radical (2^●^). The similarity of the equilibrium constant (*K*) and the rate constants involved in the equilibrium determined when oxidation of 2-SeU was induced by ^●^N_3_ at pH 6 to those when oxidation of 2-SeU was induced by ^●^OH at pH 4 (Table 1) strongly supports that in both cases, the same intermediates (6^●^ and 2^●^) are involved.

At higher pH located above the pK_a_ of 2-SeU, the initial step of the ^●^N_3_ reaction with 2-SeU is one-electron oxidation, which leads directly to the radical cation with the location of the positive charge on the selenium atom (Scheme 4).

The structures of this species and its resonance form (6^●^) are analogous to those formed from the hemibonded adduct to sulfur (1^−^ …^●^OH) (vide Scheme 2). Similarly, as in the mechanism involving ^●^OH radicals at higher concentrations of 2-SeU, radical 6^●^ is in equilibrium with the dimeric radical anion (2^●−^).

## 4. Materials and Methods

### 4.1. Chemicals

2-Selenouracil (2-SeU) was synthesized according to the published procedures [51], with a slight modification elaborated by Kulik et al. [42]. General procedure consisted of condensation of selenourea with 3-oxopropanoate, yielding 2-SeU. The resulting 2-SeU was recrystallized from ethanol and analyzed using ^1^H-, ^13^C-NMR, and mass spectrometry techniques. Sodium azide (NaN_3_) (≥99.5% purity), sodium hydroxide (NaOH) (≥99.5% purity), 70% perchloric acid (HClO_4_) (99% purity), and nitrous oxide (N_2_O) > 98% were purchased from Sigma-Aldrich (St. Louis, MO, USA) and used without further purification.

### 4.2. Preparation of Solutions

All solutions were made with deionized water (18 MΩ resistance) purified in a reverse osmosis/deionization water system from Serv-A-Pure Co (Bay City, MI, USA). The pH was adjusted by the addition of either NaOH or HClO_4_. Solutions were subsequently purged with N_2_O for at least 30 min per 200 mL of volume sample.

### 4.3. Pulse Radiolysis

Pulse radiolysis experiments with time-resolved UV-vis optical absorption detection were carried out at the Notre Dame Radiation Laboratory (NDRL), Notre Dame, Indiana, USA. The linear electron accelerator (LINAC) delivering 8 ns pulses with electron energy about 8 MeV was applied as a source of irradiation. Transient optical absorption signals were recorded in UV-visible range using multichannel system with nanosecond response recorded array of 24 monochromatic kinetic signals on all input channels of 6 synchronously triggered Tektronix oscilloscopes. The oscilloscopes were connected to an array of equivalent 24 silicon photodiode/amplifier detectors. A detailed description of the experimental setup has been given elsewhere, along with the basic details of the equipment and the continuous flow of sample solutions system [52]. Absorption intensities are presented in *G* × ε, where *G* is radiation-chemical yield (μM J^−1^) of given species and ε represents molar absorption coefficient. This value is proportional to absorbance.

The dosimetry was based on N_2_O-saturated solutions containing 10 mM KSCN, taking a radiation chemical yield of G = 0.635 μmol J^−1^ and a molar absorption coefficient of 7580 M^−1^ cm^−1^ at λ = 472 nm for the (SCN)_2_^●−^ [53]. Absorbed doses per pulse were on the order of about 15 Gy (1 Gy = 1 J kg^−1^).

The conductivity setup for time-resolved conductivity measurements was used. It allows high-precision conductometric measurements over a pH range from 3 to 6. In the current experiments, pH was restricted to 4.0. A detailed description of the conductivity setup along with the measuring cell was given elsewhere [54]. The dosimetry was achieved using acidic (pH = 4.1) aqueous solution saturated with methyl chloride (CH_3_Cl). In this dosimeter system, pulse irradiation yields H^+^ and Cl^−^ with *G*(H^+^) = *G*(Cl^−^) = 0.285 μmol J^−1^. The respective equivalent conductivities at 18 ^0^C were taken as Λ(H^+^) = 315 S cm^2^ equiv^−1^ and Λ(Cl^−^) = 65 S cm^2^ equiv^−1^ [55].

### 4.4. Theoretical Procedures

The theoretical calculations were performed using the Gaussian 16 program package [56]. Geometry optimizations, ground state reactivities, and excited-state calculations were performed using DFT range separated hybrid (RSH) functional ωB97x [57], and correlation consistent basis sets of triple ζ type, augmented with diffuse functions, denoted aug-cc-pVTZ [58,59]. Pseudopotentials given by Peterson et al. were combined with basis sets for selenium [60]. Basis Set Exchange software from the Environmental Molecular Sciences Laboratory Basis Set Library helped to generate these pseudopotentials in Gaussian format [61,62]. Application of pseudopotentials is indicated by “-PP” affix added after the name of basis sets used in the description of method in this article. The hydration effects were taken into consideration using a polarized continuum model (IEFPCM) [63]. The local minima were verified by frequency calculations. The Mulliken scheme was used to obtain spin density distribution. The genuineness of the transition states was ensured by the presence of one imaginary frequency related to either the stretching of the O–C bond (for OH addition) or H–C(N) bonds (for H abstraction) that connects the ^●^OH and 2-SeU neutral or anionic reactants. Intrinsic reaction coordinate (IRC) calculations have been carried in the vacuum out from the transition states (TS), leading to OH adducts (at S8, C2, C5, and C6 positions of neutral 2-SeU) and the pre-reactive complexes, which for some of the studied paths, started from 2c-3e OH adducts to Se atom. IRC calculations were also carried for H abstraction reactions from N3 position in neutral and anionic forms of 2-SeU. In case of anionic forms of 2-SeU, additional effort was taken to find optimized structures of pre-reactive complexes for ^●^OH addition; however, since preliminary potential energy surface scans showed lack of such complexes and their existence did not seem relevant to most of the important findings of this work, we did not pursue it further. Electronic transition energies and oscillator strengths were calculated by the time-dependent DFT (TD-DFT) method. DFT ωB97x method proven to be very satisfactory in characterization of ground-state geometries, harmonic vibrational frequencies, dissociation energies, and absorption maxima of 2c-3e intermediates of (SCN)_2_^●^ and (SeCN)_2_^●^ [46,64,65]. Similar good performance of ωB97x based TD-DFT method was documented earlier for 2-TU [66,67]. It compared well with higher levels of theory in modeling ground-state reactivity and activation barriers for ^●^OH addition to double bonds of uracil in vacuo [68], yet could differ from them in obtaining proper activation barriers in certain cases in PCM solvent and/or explicit water molecules [69]. Since ^●^OH addition leading to formation of 2c-3e SO bonds is essentially barrierless [70], applications of this relatively economic computational method served as a good compromise in quantitative estimation of relative pathways in molecular systems where H abstraction, ^●^OH addition to double bonds, and Se-O hemibond formation can occur simultaneously during oxidation of 2-SeU molecule.

## 5. Conclusions

In the current paper, we provided experimental proof supported by the density functional theory (DFT) method that the character of primary and secondary reactive intermediates depends on the concentration of 2-SeU and the pH of its aqueous solutions. Reactive intermediates observed during ^●^OH-induced oxidation of 2-SeU are mostly similar to those observed during ^●^N_3_-induced oxidation. The experiments reported here reveal some similarities and differences between 2-SeU and 2-TU. The major differences between radicals derived from them concern the stability of dimeric radicals with 2c-3e chalcogen-chalcogen bond in favor of 2-SeU and the lower yield of OH adducts to C5 = C6 double bonds in 2-SeU in comparison to 2-TU.

## Figures and Tables

**Table 1:** Equilibrium constants (*K*) and rate constants involved in equilibria.

	pH 4 (HO^●^)	pH 10 (HO^●^)	pH 6 (N_3_^●^)	pH 10 (N_3_^●^)
*K*	13,100 M^−1^	4400 M^−1^	11,900 M^−1^	3900 M^−1^
*k*_forward_	4.6 × 10^9^ M^−1^s^−1^	3.1 × 10^9^ M^−1^s^−1^	2.6 × 10^9^ M^−1^s^−1^	3.7 × 10^9^ M^−1^s^−1^
*k*_backward_	3.6 × 10^5^ s^−1 1^	7.0 × 10^5^ s^−1 1^	2.2 × 10^5^ s^−1 1^	9.5 × 10^5^ s^−1 1^

^1^ Calculated based on the equation *K* = *k*_forward_/*k*_backward_.

## Data Availability

Raw data can be obtained directly from authors.

## Supplementary Materials

The following are available online, Figure S1: Transient absorption spectra recorded in N_2_O-saturated aqueous solutions containing 1 mM of 2-SeU at pH 4 and 10, at various times after the pulse; Figure S2: Transient absorption spectra recorded in N_2_O-saturated aqueous solutions containing 30 mM NaN3 and 1 mM of 2-SeU at pH 6 and 10, at various times after the pulse; Figure S3: Absorption spectra of 2-SeU recorded in deaerated aqueous solutions at various pHs; Figure S4: Solution phase (PCM) optimized geometries of monomer-type transients expected to be formed in solutions at pH 4; Figure S5: Solution phase (PCM) optimized geometries of monomer-type transients expected to be formed in solutions at pH 10; Figure S6: Solution phase (PCM) optimized geometries of 2c-3e Se-Se dimers; Figure S7: TD-DFT calculated absorption spectra of potential transients produced in ^●^OH–induced oxidation of 2-SeU in water at pH 10; Figure S8: Relative energy profile in aqueous phase (PCM) for the •OH addition (and H abstraction) reactions to (from) 2SeU− mono-anions; Table S1: Thermochemistry values for the reactants, products, prereactive complexes, and transition states optimized structures; Table S2: Free energies of reactions of ^•^OH addition (or H abstraction) to (from) 2-SeU at pH 4 and 10. Table S3: Free energies of 2c-3e S-S dimers formation reactions.

## Sample Availability

Samples of the compounds are available from the authors.
