# Supplementary material for: Radiation-Induced Oxidation Reactions of 2-Selenouracil in Aqueous Solutions: Comparison with Sulfur Analog of Uracil
Source: Molecules. 2021 Dec 27;27(1):133. doi: 10.3390/molecules27010133 (PMC8746332; doi:10.3390/molecules27010133)
Supplement: Supplementary file 1 [file molecules-27-00133-s001.zip › molecules-1506993-supplementary.pdf]

Supplementary Material

Radiation-Induced Oxidation Reactions of 2-Selenouracil in aqueous solutions: Comparison with Sulfur Analog of Uracil

Konrad Skotnicki [1]*, Ireneusz Janik [2]*, Klaudia Sadowska [3], Grazyna Leszczynska [3], and Krzysztof Bobrowski [1]

*Correspondence : k.skotnicki@ichtj.waw.pl; Tel: +48-22-5041292; ijanik@nd.edu

Table of contents:

Figure S1	page 2
Figure S2	page 2
Figure S3	page 3
Figure S4	page 4
Figure S5	page 5
Figure S6	page 6
Figure S7	page 7
Figure S8	page 8
Table S1	page 9
Table S2	page 10
Table S3	page 11

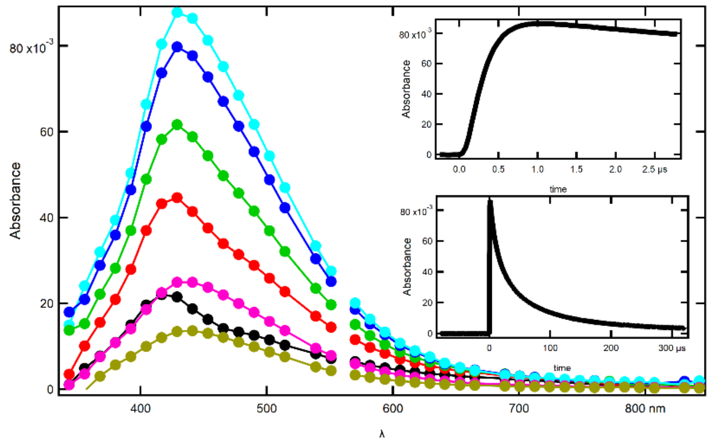

(**a**)

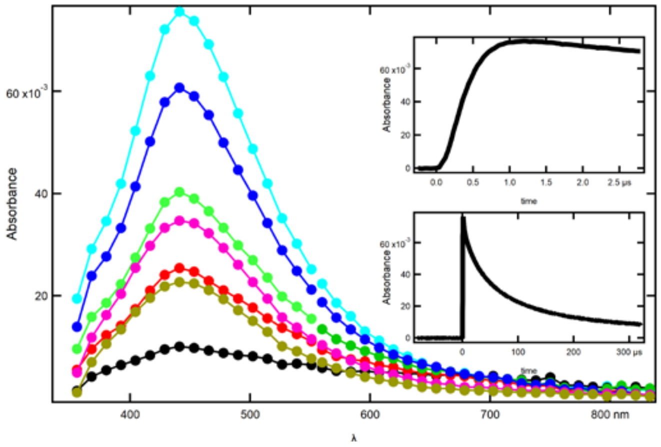

(**b**)

Figure S1. Transient absorption spectra recorded in N_2O-saturated aqueous solutions containing 1 mM of 2-SeU (**a**) at pH = 4, 100 ns (●), 200 ns (●), 300 ns (●), 500 ns (●), 1 µs (●), 50 µs (●), and 100 µs (●) after electron pulse; (**b**) at pH = 10, 100 ns (●), 200 ns (●), 300 ns (●), 500 ns (●), 1,5 µs (●), 50 µs (●), and 100 µs (●) after electron pulse. Inserts: time profiles representing growth (upper) and decay (bottom) of transient absorption at λ = 440 nm.

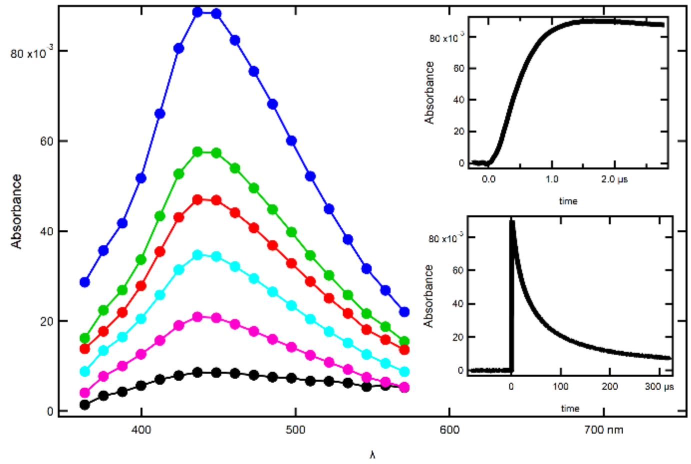

(**a**)

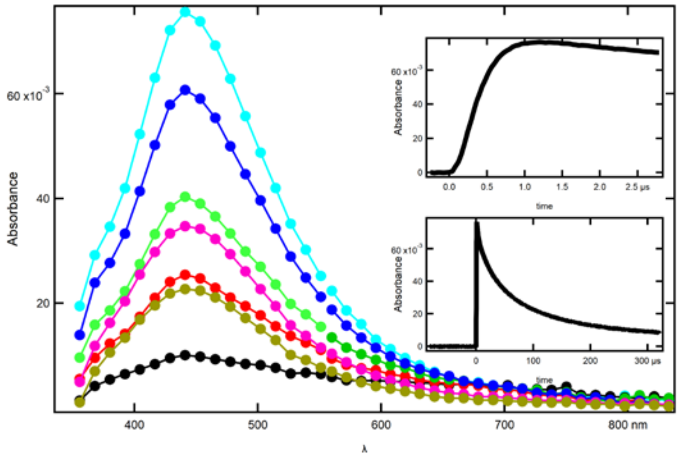

(**b**)

Figure S2. Transient absorption spectra recorded in N_2O-saturated aqueous solutions containing 30 mM NaN_3 and 1 mM of 2-SeU: (**a**) at pH 6, 100 ns (●), 400 ns (●), 500 ns (●), 1.5 µs (●), 50 µs (●), and 100 µs (●) after electron pulse; (**b**) at pH 10, 100 ns (●), 200 ns (●), 300 ns (●), 500 ns (●), 1,2 µs (●), 50 µs (●), and 100 µs (●) after electron pulse. Inserts: time profiles representing growth (upper) and decay (bottom) of transient absorption at λ = 440 nm.

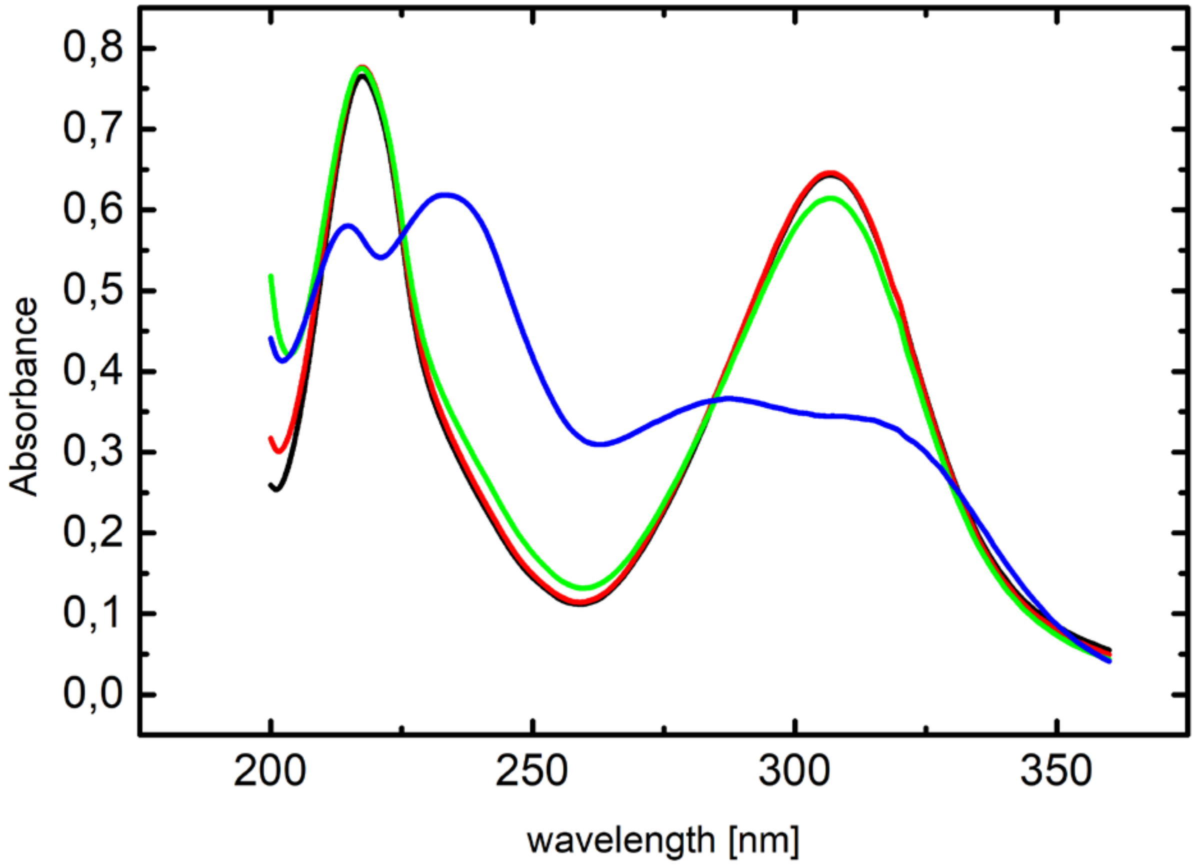

Figure S3. Absorption spectra of 2-SeU recorded in deaerated aqueous solutions at various pH: pH 3 (−), pH 5 (−), pH 6 (−), and pH 10 (−).

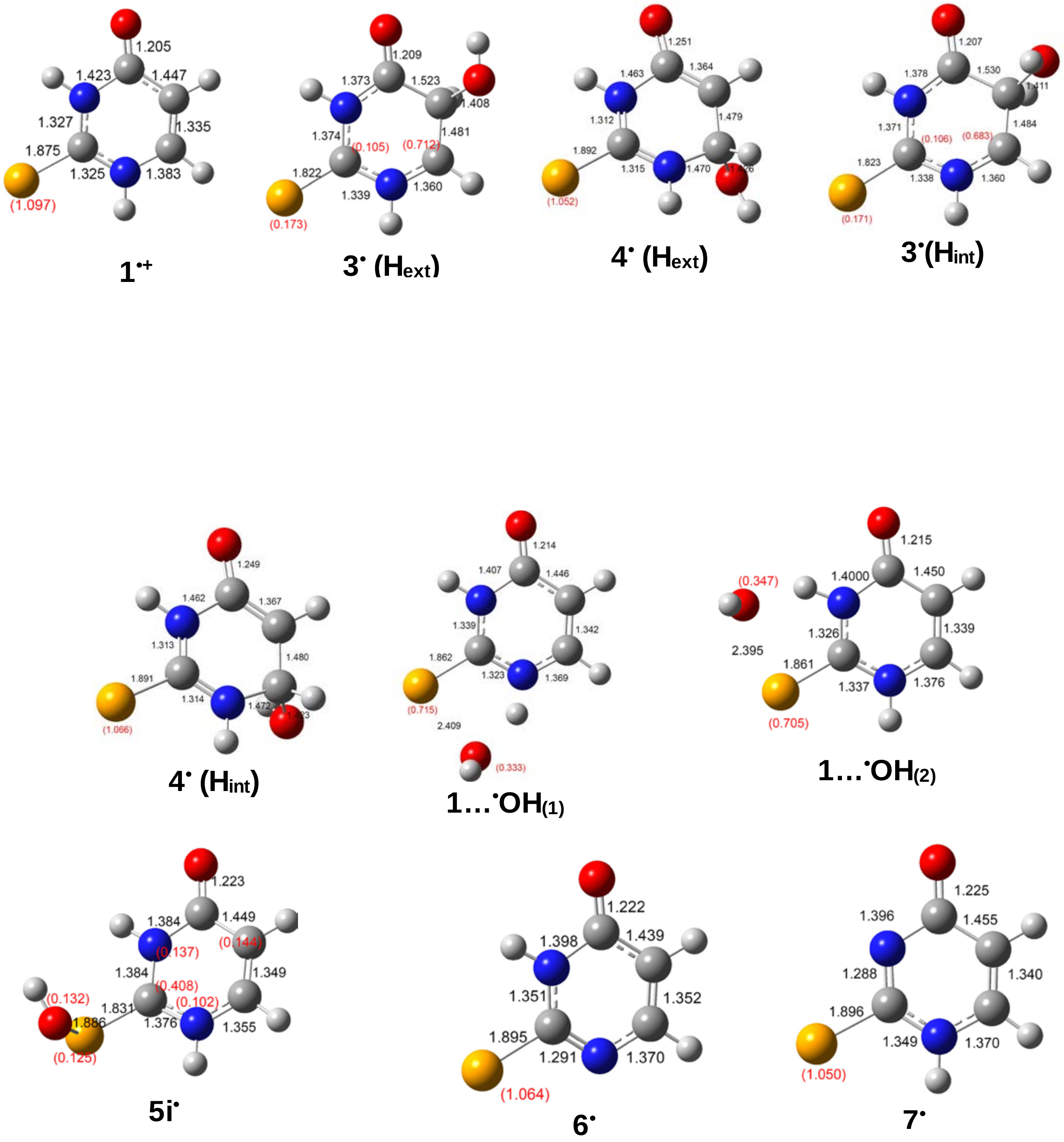

Figure S4. Solution phase (PCM) optimized geometries (ωB97x/aug-cc-pvtz) of monomer type transients expected to be formed in solutions at pH 4 (lower than the first pK_a of 2-selenouracil). Selected bond lengths are in Å units. Maximum spin population is given in red color in parentheses.

.

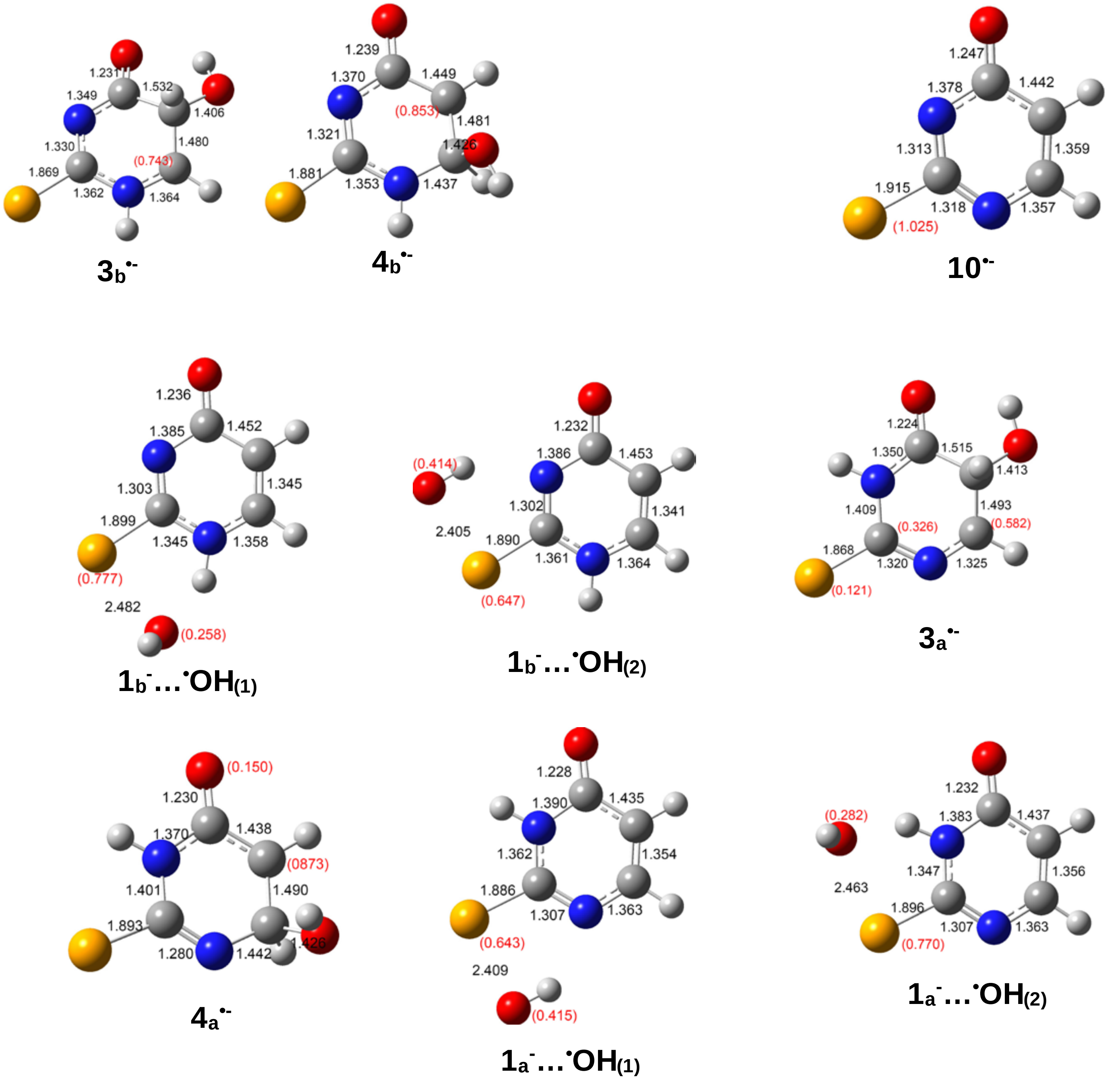

Figure S5. Solution phase (PCM) optimized geometries (ωB97x/aug-cc-pvtz-PP) of monomer type transients expected to be formed in solutions at pH 10 (higher than the first pK_a of 2-selenouracil). Selected bond lengths are in Å units. Maximum spin population is given in red color in parentheses.

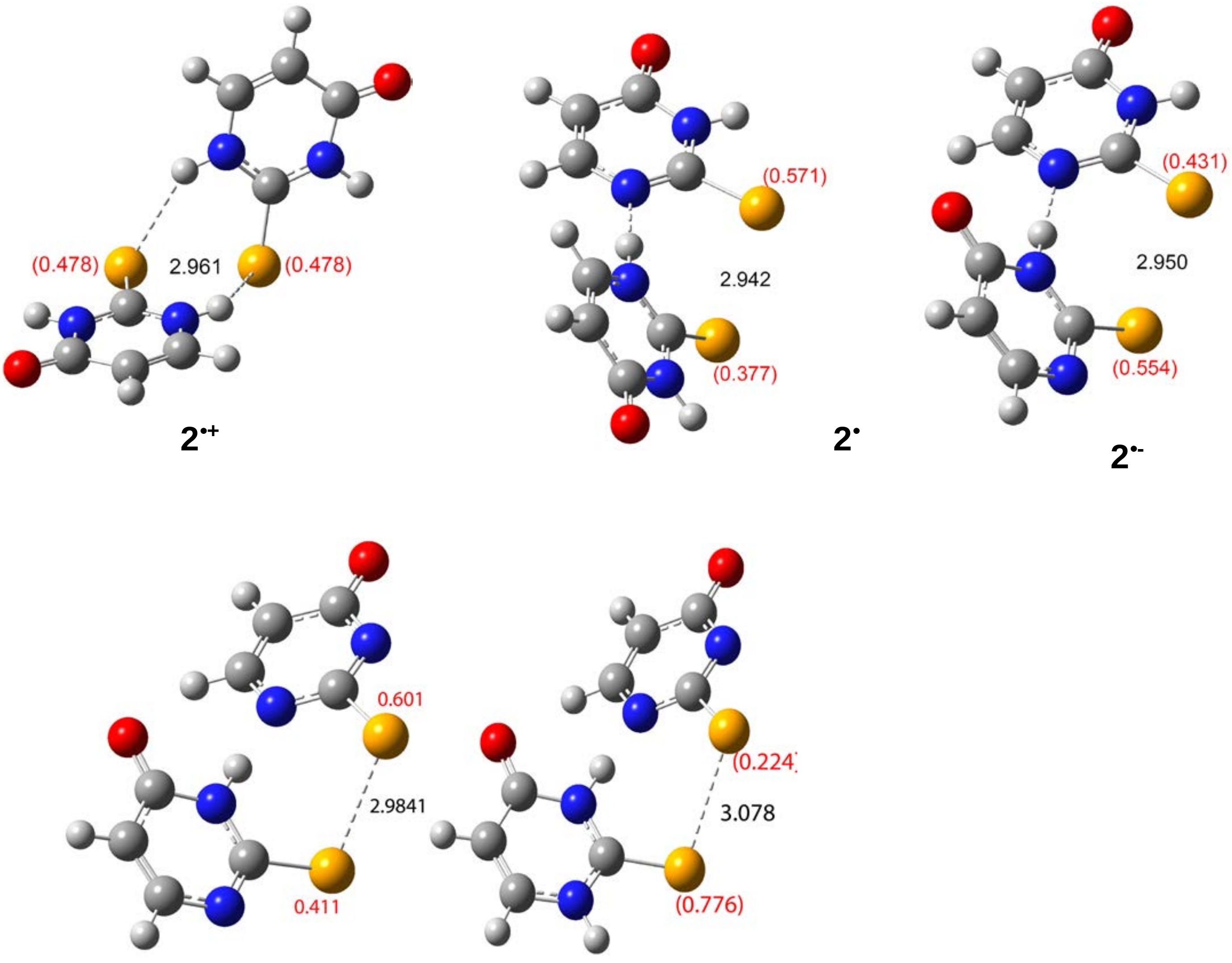

$2^{\bullet-2}$ **$2^{\bullet-}$ made of $10^{\bullet-}$ + 2-SeU**

Figure S6. Solution phase (PCM) optimized geometries (ωB97x/aug-cc-pvtz-pp) of 2c-3e SeSe dimers; SeSe bond lengths are in Å units. Maximum spin population is given in red color.

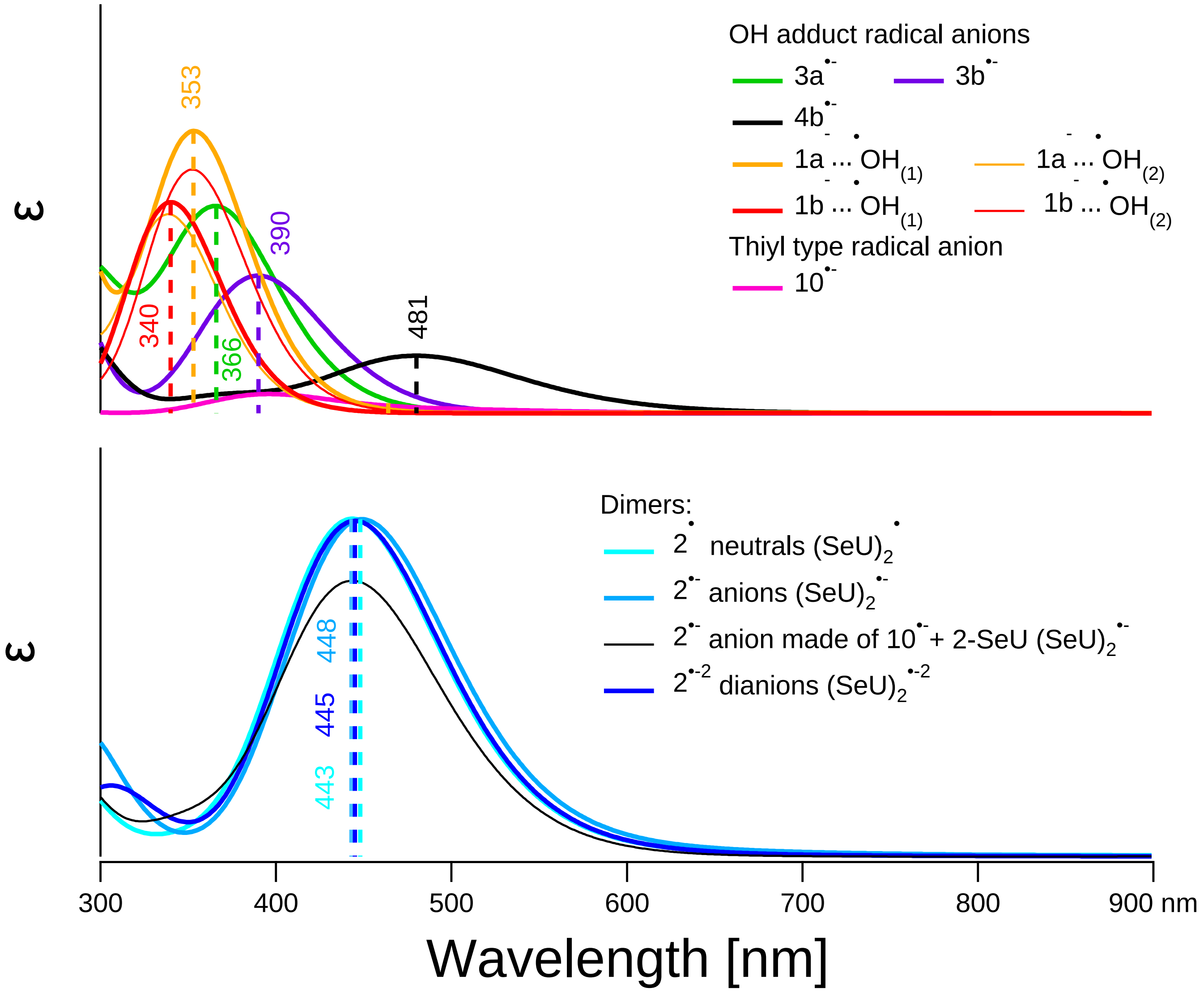

Figure S7. TD-DFT calculated absorption spectra of potential transients (see legend for symbols and Figures S5 and S6 for geometries) produced in •OH–induced oxidation of 2-selenouracil (2-SeU) in water at pH 10 (higher than its first pKa).

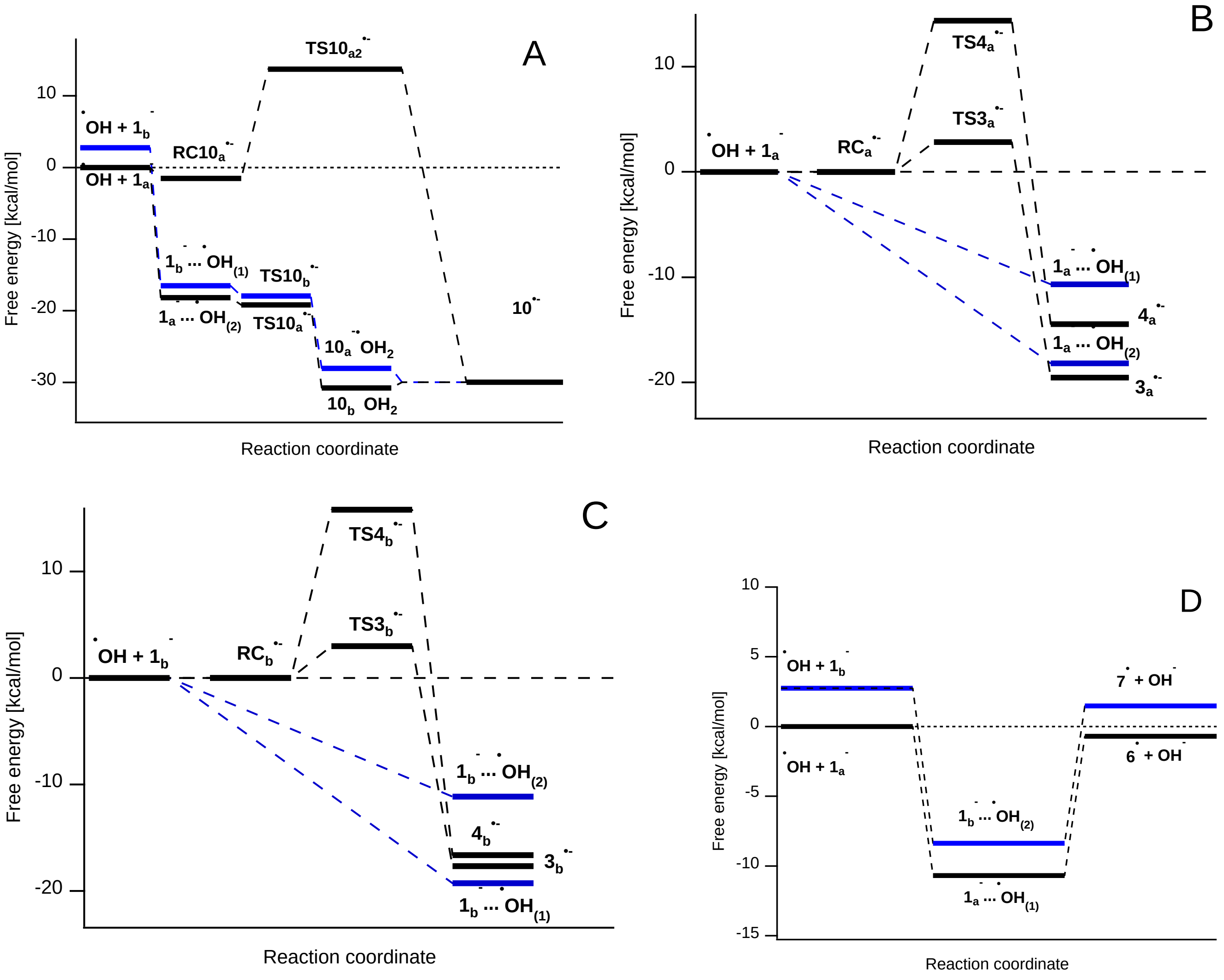

Figure S8. Relative energy profile for the H abstraction (A), •OH addition (B, C) and OH-adduct mediated electron transfer reactions (D) induced by •OH radicals reacting with 2SeU⁻ mono-anions computed in aqueous phase (PCM) at ωB97x/aug-cc-pvtz-pp level of theory.

Table S1. Thermochemistry values for the reactants, products, prereactive complexes, and transition states optimized structures at the ωB97x/aug-cc-pvtz-pp level of theory with water solvation modeled by IEFPCM. ε_0 - electronic energy, ε_{ZPE} - zero-point energy correction, E_{tot} - thermal correction to energy, H_{corr} – themal correction to enthalpy, G_{corr} - thermal correction to free energy.

Molecule	ε_0	ε_{ZPE}	E_{tot}	H_{corr}	G_{corr}	$\varepsilon_0 + \varepsilon_{ZPE}$	$\varepsilon_0 + E_{tot}$	$\varepsilon_0 + H_{corr}$	$\varepsilon_0 + G_{corr}$
					[Hartree]				
$^{\bullet}$OH	-75.744596	0.008589	0.010949	0.0119	-0.00833	-75.736007	-75.73365	-75.7327	-75.752927
H_2O	-76.448102	0.02147	0.024305	0.02525	0.00383	-76.426632	-76.423796	-76.422852	-76.444269
1	-712.866583	0.085784	0.092351	0.093295	0.05347	-712.780798	-712.77423	-712.773287	-712.813111
1^{-}_{a}	-712.402068	0.072277	0.078651	0.079595	0.04012	-712.329791	-712.32342	-712.322473	-712.361948
1^{-}_{b}	-712.397627	0.072357	0.078805	0.079749	0.04006	-712.32527	-712.31882	-712.317878	-712.357564
$1^{\bullet+}$	-712.645392	0.085516	0.092359	0.093304	0.05216	-712.559876	-712.55303	-712.552088	-712.593233
$2^{\bullet+}$	-1425.54439	0.172785	0.18791	0.188854	0.12519	-1425.3716	-1425.3565	-1425.355534	-1425.419196
$2^{\bullet}$	-1425.10449	0.159074	0.173825	0.174769	0.11266	-1424.94542	-1424.9307	-1424.929722	-1424.991835
$2^{\bullet-}$	-1424.64678	0.145869	0.160503	0.161448	0.09925	-1424.50091	-1424.4863	-1424.485329	-1424.547531
$2^{\bullet-2}$	-1424.166674	0.132078	0.146565	0.147509	0.085136	-1424.0346	-1424.0201	-1424.019165	-1424.081538
$3^{\bullet}$ (H_{ext})	-788.658044	0.099404	0.108003	0.108947	0.06356	-788.558641	-788.55004	-788.549097	-788.594482
$3^{\bullet}$ (H_{int})	-788.656885	0.099871	0.108293	0.109237	0.0644	-788.557014	-788.54859	-788.547648	-788.592486
$4^{\bullet}$ (H_{ext})	-788.627879	0.100095	0.108375	0.109319	0.0649	-788.527783	-788.5195	-788.518559	-788.562983
$4^{\bullet}$ (H_{int})	-788.630209	0.10022	0.108453	0.109397	0.06511	-788.529989	-788.52176	-788.520812	-788.565098
1…$^{\bullet}OH_{(1)}$	-788.650443	0.097078	0.105766	0.10671	0.06173	-788.553365	-788.54468	-788.543733	-788.588716
1…$^{\bullet}OH_{(2)}$	-788.649083	0.097402	0.106132	0.107076	0.06201	-788.551681	-788.54295	-788.542006	-788.587074
1_a^{-}…$^{\bullet}OH_{(1)}$	-788.180375	0.084565	0.093397	0.094342	0.04848	-788.09581	-788.08698	-788.086034	-788.131899
1_a^{-}…$^{\bullet}OH_{(2)}$	-788.19236	0.083929	0.09259	0.093534	0.04851	-788.108431	-788.09977	-788.098826	-788.143851
1_b^{-}…$^{\bullet}OH_{(1)}$	-788.189568	0.083845	0.0925	0.093444	0.04836	-788.105724	-788.09707	-788.096124	-788.141205
1_b^{-}…$^{\bullet}OH_{(2)}$	-788.176302	0.084628	0.093531	0.094476	0.04808	-788.091674	-788.08277	-788.081826	-788.128221
$3_a^{\bullet-}$	-788.196474	0.085895	0.09416	0.095104	0.05046	-788.110578	-788.10231	-788.101369	-788.146014
$3_b^{\bullet-}$	-788.189759	0.086003	0.094131	0.095076	0.05114	-788.103757	-788.09563	-788.094684	-788.138621
$4_a^{\bullet-}$	-788.188049	0.085946	0.094336	0.09528	0.05013	-788.102103	-788.09371	-788.092769	-788.137917
$4_b^{\bullet-}$	-788.187253	0.085996	0.094407	0.095351	0.05028	-788.101256	-788.09285	-788.091902	-788.136976
$6^{\bullet}$	-712.210618	0.071546	0.077646	0.078591	0.0389	-712.139072	-712.13297	-712.132028	-712.171717
$7^{\bullet}$	-712.207269	0.072315	0.078974	0.079918	0.03902	-712.134954	-712.1283	-712.127351	-712.168254
$9^{\bullet}$	-788.660691	0.09923	0.107742	0.108686	0.06408	-788.561461	-788.55295	-788.552005	-788.596611
$10^{\bullet-}$	-711.744256	0.058892	0.065284	0.066228	0.02587	-711.685364	-711.67897	-711.678028	-711.718391
$RC^{\bullet}$	-788.620666	0.096204	0.106009	0.106953	0.05839	-788.524462	-788.51466	-788.513713	-788.562279
$TS3^{\bullet}$	-788.620173	0.096433	0.105301	0.106245	0.06016	-788.523741	-788.51487	-788.513929	-788.560012
$TS4^{\bullet}$	-788.618155	0.09676	0.105291	0.106235	0.06123	-788.521395	-788.51286	-788.51192	-788.556924
$RC7^{\bullet}$	-788.625342	0.097038	0.106095	0.107039	0.06018	-788.528304	-788.51925	-788.518302	-788.565164
$TS3a^{\bullet-}$	-788.157692	0.083074	0.091489	0.092433	0.04733	-788.074618	-788.0662	-788.065259	-788.110361
$TS4a^{\bullet-}$	-788.13856	0.082528	0.091219	0.092163	0.04656	-788.056031	-788.04734	-788.046396	-788.091998
$TS3b^{\bullet-}$	-788.152904	0.083172	0.09175	0.092694	0.04719	-788.069732	-788.06115	-788.06021	-788.105716
$TS4b^{\bullet-}$	-788.136516	0.082733	0.091417	0.092361	0.04682	-788.053783	-788.0451	-788.044155	-788.089693
$TS6^{\bullet}$	-788.65009	0.094349	0.102543	0.103487	0.0595	-788.555741	-788.54755	-788.546603	-788.590595
$TS7^{\bullet}$	-788.648114	0.094009	0.102295	0.103239	0.05905	-788.554105	-788.54582	-788.544875	-788.589063
$TS10a^{\bullet-}$	-788.190194	0.079846	0.088139	0.089083	0.04476	-788.110348	-788.1021	-788.10111	-813.06482
$TS10b^{\bullet-}$	-788.188418	0.080089	0.08837	0.089314	0.04497	-788.10833	-788.10005	-788.099105	-788.143452

Table S2. Free energies of reactions of $^{\bullet}OH$ addition, H abstraction, and OH-adduct mediated electron transfer induced by $^{\bullet}OH$ radical reacting with 2-SeU at pH 4 and 10.

Reaction	$\Sigma(\varepsilon_0 + G_{corr})$ [Hartree]		Δ_rG^0(298K) [kcal/mol] values for analogous 2-TU intermediates in parentheses
	Reactants	Products	
$^{\bullet}OH + 1 \rightarrow 1...^{\bullet}OH_{(1)}$	-788.566038	-788.589	-14.23 (-6.60)
$^{\bullet}OH + 1 \rightarrow 1...^{\bullet}OH_{(2)}$	-788.566038	-788.587	-13.20 (-5.95)
$^{\bullet}OH + 1 \rightarrow 6^{\bullet} + H_2O$	-788.566038	-788.615986	-31.34 (-23.16)
$^{\bullet}OH + 1 \rightarrow 7^{\bullet} + H_2O$	-788.566038	-788.612523	-29.17 (-18.61)
$^{\bullet}OH + 1 \rightarrow 9^{\bullet} + H_2O$	-788.566038	-788.596611	-19.18 (n.d.)
$^{\bullet}OH + 1 \rightarrow TS6^{\bullet}$	-788.566038	-788.143452	-15.41 (-6.91)
$^{\bullet}OH + 1 \rightarrow TS7^{\bullet}$	-788.566038	-788.589063	-14.45 (-5.6)
$^{\bullet}OH + 1 \rightarrow RC7^{\bullet}$	-788.566038	-788.565164	0.55 (0.42)
$^{\bullet}OH + 1 \rightarrow TS9^{\bullet}$	-788.566038	-788.567252	-14.45 (n.d.)
$^{\bullet}OH + 1 \rightarrow 3^{\bullet}$ (H_{ext})	-788.566038	-788.594	**-17.85** (-17.47)
$^{\bullet}OH + 1 \rightarrow 3^{\bullet}$ (H_{int})	-788.566038	-788.592	-16.60 (-16.40)
$^{\bullet}OH + 1 \rightarrow 4^{\bullet}$ (H_{ext})	-788.566038	-788.563	1.92 (-19.23)
$^{\bullet}OH + 1 \rightarrow 4^{\bullet}$ (H_{int})	-788.566038	-788.565	**0.59** (-19.37)
$^{\bullet}OH + 1 \rightarrow RC^{\bullet}$	-788.566038	-788.562	2.36 (2.09)
$^{\bullet}OH + 1 \rightarrow TS3^{\bullet}$	-788.566038	-788.56	3.78 (3.52)
$^{\bullet}OH + 1 \rightarrow TS4^{\bullet}$	-788.566038	-788.557	5.72 (5.56)
$^{\bullet}OH + 1^{-}_{a} \rightarrow 1_a^{-}...^{\bullet}OH_{(1)}$	-788.114875	-788.132	-10.68 (-5.99)
$^{\bullet}OH + 1^{-}_{a} \rightarrow 1_a^{-}...^{\bullet}OH_{(2)}$	-788.114875	-788.144	-18.18 (-11.20)
$^{\bullet}OH + 1^{-}_{b} \rightarrow 1_b^{-}...^{\bullet}OH_{(1)}$	-788.110491	-788.141	-19.27 (-12.23)
$^{\bullet}OH + 1^{-}_{b} \rightarrow 1_b^{-}...^{\bullet}OH_{(2)}$	-788.110491	-788.128	-11.12 (-6.49)
$^{\bullet}OH + 1^{-}_{a} \rightarrow 10^{\bullet-} + H_2O$	-788.114875	-788.16266	-29.98 (-22.32)
$^{\bullet}OH + 1^{-}_{b} \rightarrow 10^{\bullet-} + H_2O$	-788.110491	-788.16266	-32.73 (-25.17)
$^{\bullet}OH + 1^{-}_{a} \rightarrow TS10_a^{\bullet-}$	-788.114875	-788.145433	-19.17 (13.61)
$^{\bullet}OH + 1^{-}_{b} \rightarrow TS10_b^{\bullet-}$	-788.110491	-788.143452	-20.68 (-12.7)
$^{\bullet}OH + 1^{-}_{a} \rightarrow 3_a^{\bullet-}$	-788.114875	-788.146	-19.54 (-19.12)
$^{\bullet}OH + 1^{-}_{a} \rightarrow 4_a^{\bullet-}$	-788.114875	-788.138	-14.46 (-13.89)
$^{\bullet}OH + 1^{-}_{b} \rightarrow 3_b^{\bullet-}$	-788.110491	-788.139	-17.65 (-17.89)
$^{\bullet}OH + 1^{-}_{b} \rightarrow 4_b^{\bullet-}$	-788.110491	-788.137	-16.62 (-16.77)
$^{\bullet}OH + 1^{-}_{a} \rightarrow TS3_a^{\bullet-}$	-788.114875	-788.11	2.83 (2.69)
$^{\bullet}OH + 1^{-}_{a} \rightarrow TS4_a^{\bullet-}$	-788.114875	-788.092	14.35 (4.86)
$^{\bullet}OH + 1^{-}_{b} \rightarrow TS3_b^{\bullet-}$	-788.110491	-788.106	2.99 (2.91)
$^{\bullet}OH + 1^{-}_{b} \rightarrow TS4_b^{\bullet-}$	-788.110491	-788.09	15.80 (6.65)
$1_a^{-}...^{\bullet}OH_{(1)} \rightarrow 6^{\bullet} + OH^{-}$	-788.1319	-788.1160	9.98 (12.02)
$1_b^{-}...^{\bullet}OH_{(2)} \rightarrow 7^{\bullet} + OH^{-}$	-788.1282	-788.1125	9.85 (14.21)

Table S3. Free energies of 2c-3e S-S dimers formation reactions induced by •OH and •N_3 radicals reacting with 2-SeU at pH 4 and 10 and pH 6 and 10, respectively.

Reaction	$\Sigma(\varepsilon_0 + G_{corr})$ [Hartree]		$\Delta_r G^0$(298K) [kcal/mol] values for analogous 2-TU intermediates in parentheses
	Reactants	Products	
**$1 + 1^{\bullet+} \rightarrow 2^{\bullet+}$**	-1425.4063	-1425.4192	-8.06 (-7.50)
**$1 + 6^{\bullet} \rightarrow 2^{\bullet}$**	-1424.9848	-1424.9918	-4.40 (-0.55)
**$1_a^- + 6^{\bullet} \rightarrow 2^{\bullet -}$**	-1424.5337	-1424.5475	-8.70 (-6.04)
**$1a^- + 10^{\bullet -} \rightarrow 2^{\bullet -2}$**	-1424.0760	-1424.0815	-3.50 (n.d.)
**$1 + 10^{\bullet -} \rightarrow 2^{\bullet -}$**	1424.5315	1424.533	-0.9260 (n.d.)
